# Epigenetics and childhood obesity: DNA methylation coordinates environment and gene regulation

**DOI:** 10.1210/endrev/bnag013

**Published:** 2026-05-11

**Authors:** Suneesh Kaimala, Melvin Khee-Shing Leow, Suraiya Anjum Ansari, Bright Starling Emerald

**Affiliations:** Department of Anatomy, College of Medicine and Health Sciences, United Arab Emirates University, Al Ain, Abu Dhabi 15551, United Arab Emirates; Institute for Human Development and Potential (IHDP), Agency for Science, Technology and Research (A*STAR), 30 Medical Drive, Singapore 117609, Republic of Singapore; Singapore Institute of Food and Biotechnology Innovation, A*STAR, Singapore 138634, Republic of Singapore; Department of Endocrinology, Tan Tock Seng Hospital, Singapore 308433, Republic of Singapore; Cardiovascular and Metabolic Diseases Program, Duke-NUS Medical School, Singapore 169857, Republic of Singapore; Lee Kong Chian School of Medicine, Nanyang Technological University, Singapore 308232, Republic of Singapore; Department of Biochemistry and Molecular Biology, College of Medicine and Health Sciences, United Arab Emirates University, Al Ain, Abu Dhabi 15551, United Arab Emirates; Zayed Center for Health Sciences, United Arab Emirates University, Al Ain, Abu Dhabi 15551, United Arab Emirates; ASPIRE Precision Medicine, Research Institute Abu Dhabi, Al Ain, Abu Dhabi 15551, United Arab Emirates; Department of Anatomy, College of Medicine and Health Sciences, United Arab Emirates University, Al Ain, Abu Dhabi 15551, United Arab Emirates; Zayed Center for Health Sciences, United Arab Emirates University, Al Ain, Abu Dhabi 15551, United Arab Emirates; ASPIRE Precision Medicine, Research Institute Abu Dhabi, Al Ain, Abu Dhabi 15551, United Arab Emirates

**Keywords:** childhood obesity, epigenetic regulation, DNA methylation, early environment, gene expression

## Abstract

Childhood obesity is a complex disorder which results from the combined contribution of genetics, the environment, and development, which is programmed and coordinated by epigenetic mechanisms. Of them, DNA methylation has emerged as an important molecular interface between environmental inputs and changes in gene expression. In this review, we provide an overview of the role of DNA methylation in childhood obesity during the key developmental stages, from prenatal life and childhood to adolescence. We also highlight the available evidence from candidate genes and genome-wide association studies implicating critical loci involved in energy homeostasis and adipogenesis, where DNA methylation is altered. Further, we also provide an overview of how maternal obesity, nutritional status, and bariatric surgery shape offspring's methylation profiles and contribute to the increased risk of programming obesity across generations.

Although aberrant methylation patterns are consistently associated with altered metabolic phenotypes, disentangling causality remains a significant challenge. Herein, we highlight emerging approaches, such as rigorous longitudinal cohorts, epigenetic Mendelian randomization, and CRISPR-based epigenome editing, that are beginning to provide the analytical clarity needed to move beyond association.

Finally, we examine the potential of DNA methylation signatures to inform early risk stratification and prevention possibilities. Although yet to be clinically validated, whole-genome methylation profiling is increasingly integrated with systems biology and multi-omics frameworks, making the identification of robust, clinically actionable markers more promising. A more precise understanding of how epigenetic processes shape susceptibility to childhood obesity could ultimately support strategies capable of altering lifelong metabolic trajectories.

## Essential points

- Epigenetic marks, such as DNA methylation established during prenatal and early postnatal development integrate maternal metabolic status, nutrition, and environmental exposures, shape gene expression programs, which in turn influence growth trajectories and metabolic health, including obesity across the life course
- Maternal metabolic health, such as maternal obesity, glycemic status, dietary quality, and metabolic interventions, exerts long-lasting effects on the offspring's epigenome through methylation changes
- While DNA methylation in candidate genes such as *LEP*, *POMC*, *IGF2*, *HIF3A*, and *PPARGC1A* provides mechanistic insight into metabolic regulatory changes of childhood obesity, epigenome-wide methylation studies highlight broader involvement in childhood obesity susceptibility
- Many methylation changes reflect shared genetics, correlational alterations, or postnatal exposures rather than direct causal mechanisms, underscoring the need for integrative approaches combining longitudinal cohorts, genetic causal inference, and functional epigenome editing
- Methylation patterns detectable at birth and during adolescence may support early risk stratification, while emerging intervention studies demonstrate that selected epigenetic marks are modifiable, providing a foundation for personalized prevention strategies aimed at reducing childhood obesity risk

## Introduction

The prevalence of obesity, a multifactorial disease, has become the new pandemic over the past 5 decades and has emerged as an important public health issue. It is characterized by excess adiposity and a complex etiology that involves genetic, environmental, behavioral, and socioeconomic determinants. Among adults, the prevalence of obesity has risen markedly, nearly tripling in women (from 6.6% to 18.5%) and quadrupling in men (from 3.0% to 14.0%) between 1975 and 2022. On the other hand, childhood and adolescent obesity rates have increased approximately 10-fold, and in 2022, 9.3% of boys and 6.9% of girls aged 5 to 19 years were shown to live with obesity, corresponding to an estimated 159 million school-aged children and adolescents worldwide (1).

**Figure 1:**
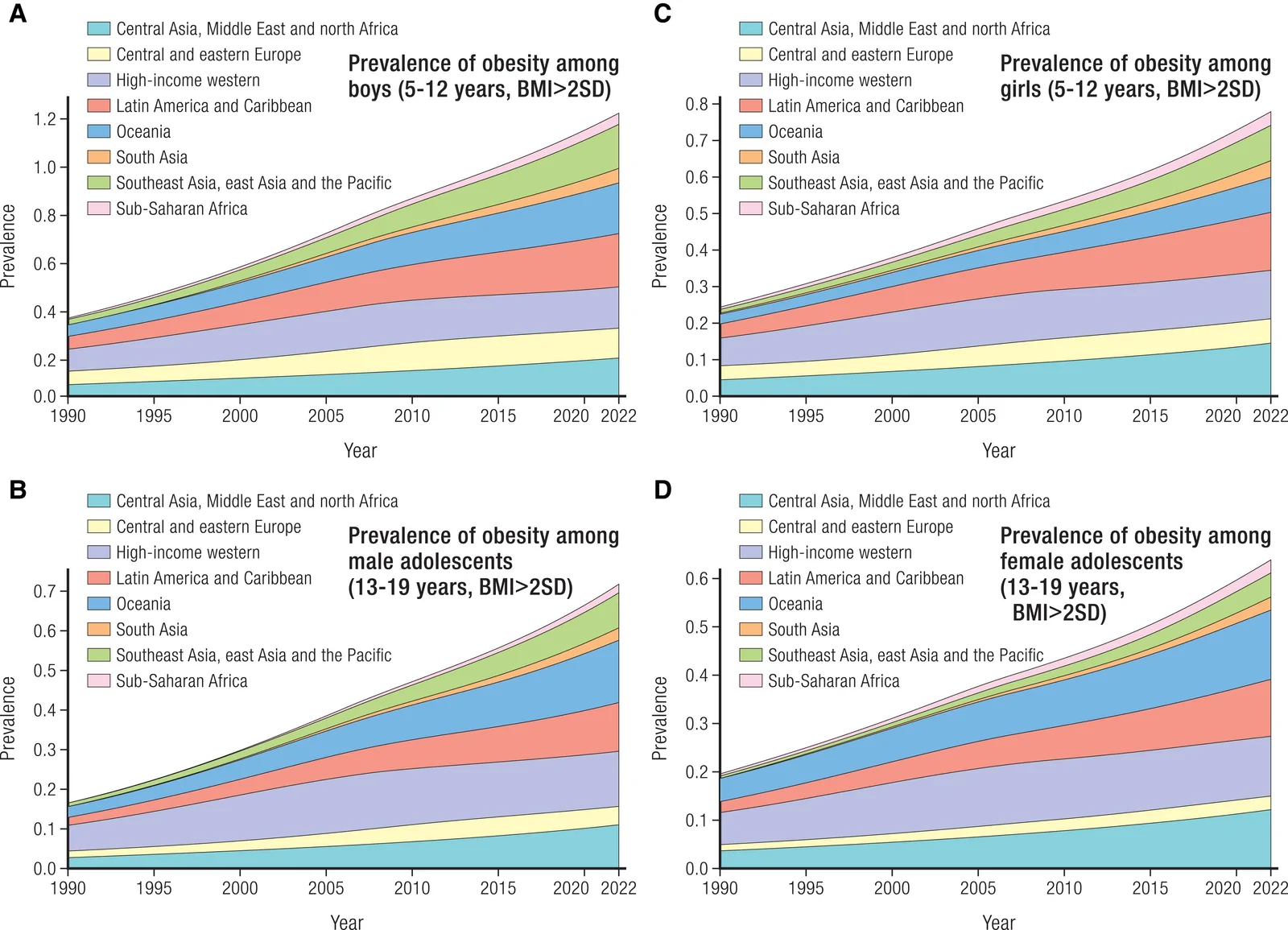
Global prevalence of childhood obesity by sex, age group, and geographical region. The global burden of childhood obesity has increased markedly over recent decades across diverse populations. (A and B) Prevalence of obesity (defined as weight ≥ 2 s.d. above the median of the WHO growth reference) among male children aged 5 to 12 years and male adolescents aged 13 to 19 years from 1990 to 2022. (C and D) Corresponding prevalence estimates for female children aged 5 to 12 years and female adolescents aged 13 to 19 years over the same period. Distinct colors indicate geographical regions. Graphs were generated using the data from NCD Risk Factor Collaboration (NCD RisC) platform and Ref. (5).

This condition is further exacerbated by the World Health Organization report, which estimated that around 35 million children under the age of 5 years were overweight, underscoring the growing burden of early-life obesity. It is also true that, once considered a high-income country problem predominantly, overweight and obesity are now rapidly rising in low- and middle-income countries. In Africa, the number of overweight children under 5 years of age has increased by nearly 12.1% since 2000, while almost half of all children under 5 years of age with overweight or obesity in 2024 were living in Asia. Among older age groups, in 2022, more than 390 million children and adolescents aged 5 to 19 years were overweight. The global prevalence of overweight (including obesity) in this age group has increased from 8% in 1990 to 20% in 2022, with comparable patterns in both sexes (19% in girls and 21% in boys). The prevalence of obesity alone has risen 4-fold during the same period, from 2% (31 million) in 1990 to 8% (160 million) in 2022 (www.who.int/news-room/fact-sheets/detail/obesity-and-overweight). Childhood obesity substantially increases the risk of lifelong obesity and accelerates the onset of associated metabolic and psychiatric comorbidities, including type 2 diabetes, cardiovascular disease, and mood disorders (2, 3). Furthermore, obesity reduces health-adjusted life expectancy and contributes significantly to disability-adjusted life years lost worldwide (Fig. 1) (4).

Obesity is predominantly a multifactorial condition resulting from the interplay between genetic predisposition and environmental exposures. Thus, traditional genetic models do not explain the rapid increase in obesity prevalence. Epigenetics provides a molecular framework for understanding how external factors, such as the environment, can induce heritable but reversible modifications in gene expression without altering the DNA sequence, thus linking early developmental environment and lifestyle exposures to phenotypic outcomes. Hence, evidence-based and multisectoral strategies are needed to address the increasing burden of obesity and its associated consequences, which cause health issues throughout the lifetime (1). The interaction between genetic susceptibility and exposures to different environments with variations that cause changes in the epigenetic regulatory mechanisms introduces substantial interindividual variability in growth, including weight trajectories. This interaction, while reflecting the complex and dynamic biological architecture which underlies the mechanism of obesity, also highlights why populations and individuals respond differently to similar environmental exposures and a possible explanation for why metabolic risk unfolds along divergent developmental pathways (6, 7).

## Epigenetic regulation in obesity

### Molecular basis of epigenetic regulation

**Figure 2:**
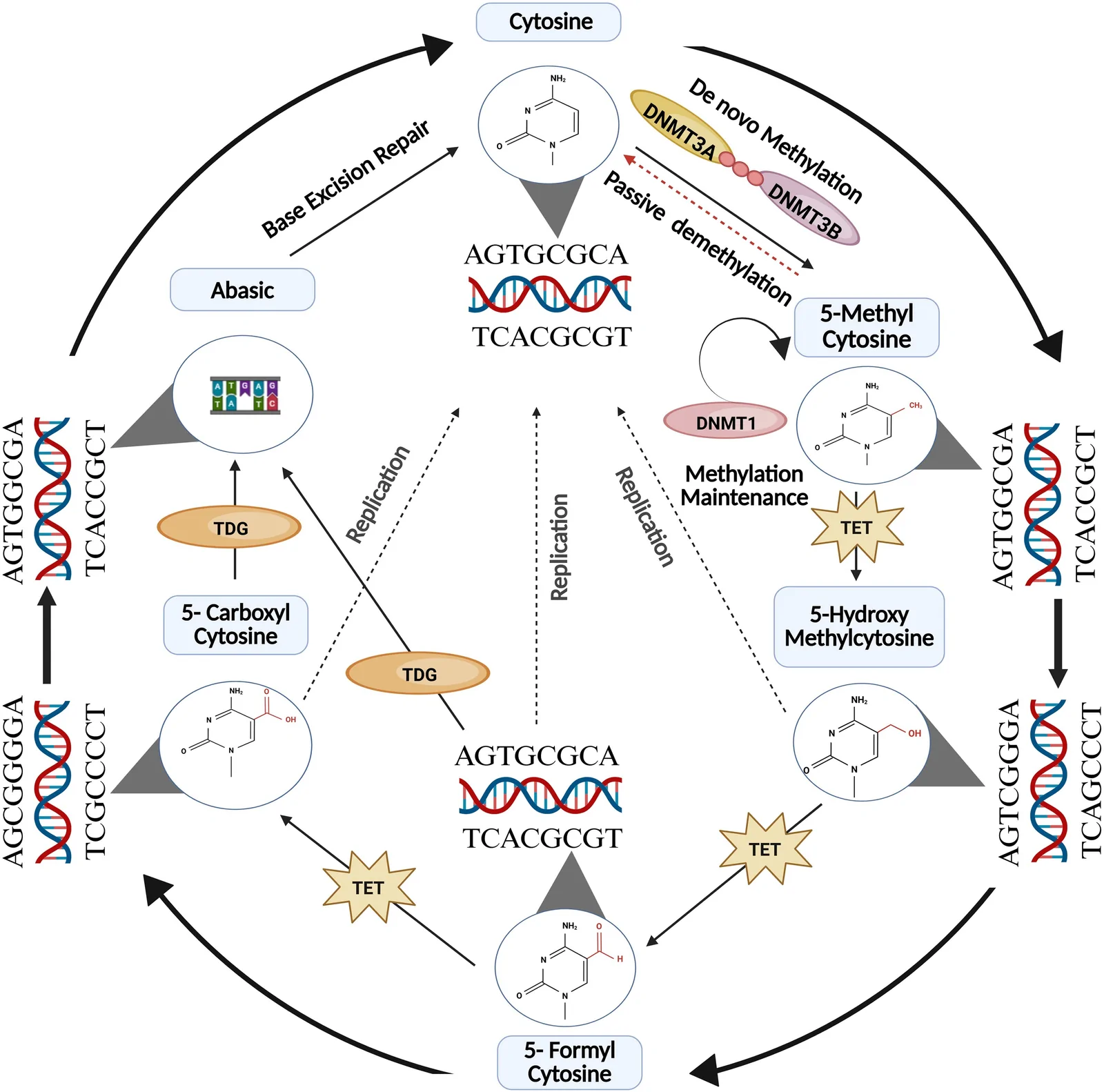
DNA methylation and active vs passive mechanisms of DNA demethylation. DNA methylation involves the addition of a methyl group (CH_3_) to cytosine within CpG dinucleotides by DNA methyltransferases (DNMTs), generating 5-methylcytosine (5mC). Active demethylation is mediated by TET dioxygenases, which sequentially oxidize 5mC to 5-hydroxymethylcytosine (5hmC), 5-formylcytosine (5fC), and 5-carboxylcytosine (5caC), followed by base excision repair that restores unmethylated cytosine. Passive demethylation arises when methylated sites fail to be remethylated during DNA replication, leading to progressive dilution of 5mC and reestablishment of unmodified cytosine. Abbreviations: DNMT1, DNA methyltransferase; 1DNMT3a, DNA methyltransferase 3 Alpha; DNMT3b, DNA methyltransferase 3 Beta; TDG, thymine DNA glycosylase; TET, Tet methylcytosine dioxygenase. Figure generated using BioRender.

Epigenetic regulation includes DNA methylation, histone modifications, and noncoding RNA-mediated processes, each of which contributes to chromatin availability and fine-tuning of gene expression. DNA methylation involves the covalent addition of a methyl group to the fifth carbon of cytosine (5mC), predominantly within CpG dinucleotides, catalyzed by the DNA methyltransferases (DNMTs), namely, DNMT1 (maintenance DNA methyltransferase), DNMT3A, and DNMT3B (de novo DNA methyltransferases) (8). DNA methylation typically promotes a repressive chromatin state by recruiting methyl-CpG-binding domain proteins (MBD1-3) and associated histone-modifying and corepressor complexes, which collectively compact chromatin and result in the modulation of transcriptional activity (Fig. 2) (9, 10).

Demethylation of 5-methylcytosine occurs via 2 mechanistically distinct pathways, namely, the passive and the active demethylation pathways. Passive demethylation occurs during DNA replication when DNMT1-mediated maintenance methylation is reduced or absent, which results in the gradual dilution of methylation marks across successive cell divisions (11). In contrast, active DNA demethylation is driven by an enzyme-driven process and is replication independent. The enzymes that mediate this process primarily consist of the ten-eleven translocation (TET) family of dioxygenases, ten-eleven translocation methylcytosine dioxygenase 1 (TET1), ten-eleven translocation methylcytosine dioxygenase 2 (TET2), and ten-eleven translocation methylcytosine dioxygenase 3 (TET3). These enzymes catalyze the iterative oxidation of 5mC to 5-hydroxymethylcytosine (5hmC), then to 5-formylcytosine (5fC), and finally to 5-carboxylcytosine (5caC) (12, 13). The enzyme thymine DNA glycosylase (TDG) then excises the oxidized cytosine derivatives, which are replaced with unmodified cytosines through the base excision repair (BER) pathway, thus restoring unmethylated cytosine residues (Fig. 2) (11, 13, 14).

Dynamic DNA methylation and demethylation regulation are an integral part of the epigenomic plasticity, which plays crucial roles in cellular differentiation, embryonic development, and environmental adaptation. Aberrations in these epigenetic processes contribute to widespread dysregulation in the regulatory mechanisms and have been shown to be associated with a variety of disease states, including oncogenesis, neurodevelopmental disorders, and metabolic diseases such as obesity, cardiovascular disease, and type 2 diabetes. In metabolic diseases, altered DNA methylation patterns have been shown to disrupt gene regulatory networks that coordinate and control energy homeostasis, adipogenesis, and insulin sensitivity, thus contributing to the development and progression of metabolic diseases, including obesity (15-17).

Histones, the group of basic proteins to which DNA wraps around, and for the structural units, also undergo various types of posttranslational modification, including acetylation and methylation, which represent another epigenetic layer modulated by numerous enzymes that dynamically regulate the structure and gene activity (18). Long noncoding RNAs also contribute to epigenetic regulation by affecting DNA methylation or histone modifications (19, 20). Alterations in epigenetic architecture result in changes in critical regulatory pathways, which have been shown to influence childhood obesity. In this review, we will focus primarily on the role of DNA methylation and its contributions to the development of childhood obesity.

### Prenatal environmental factors and DNA methylation signatures of adiposity in offspring

Maternal exposure to environmental stressors during gestation can alter the epigenomes of the offspring through DNA methylation changes, influencing genes vital for development and susceptibility to long-term diseases (21). Some of the maternal or developmental environmental factors that affect the pattern of fetal methylation are maternal hyperglycemia, maternal high body mass index (BMI), maternal preeclampsia, maternal nutrition, and lifestyle.

### Maternal hyperglycemia

Maternal hyperglycemia during gestation, as seen in gestational diabetes, has been shown to induce methylation changes in the fetus, predisposing to metabolic, cardiovascular, and neurodevelopmental disorders later in life (21). Leptin, encoded by the LEP gene, plays a central role in appetite regulation and energy homeostasis by signaling satiety to the brain and modulating fat storage. Epigenetic regulation of LEP through DNA methylation has emerged as a key determinant of leptin expression. In rodent models, hypermethylation of the Lep promoter suppresses transcription, reduces the abundance of leptin mRNA, and contributes to leptin resistance, thereby impairing hypothalamic appetite regulation in obesity (22). Translating these mechanistic insights, during human pregnancy, maternal glycemic status has been shown to influence LEP methylation of the developing fetus. By using a 2-step epigenetic Mendelian randomization framework, maternal fasting glucose levels quantified by a 10-variant glycemia risk score (GRS10) were shown to be associated with reduced methylation levels at a LEP-linked CpG site (cg12083122) in offspring. The lower methylation levels at this locus corresponded to elevated cord-blood leptin concentrations and established a plausible causal connection between maternal glycemia, fetal epigenetic remodeling, and early leptin signaling (23). In parallel, a rat experimental model showed that a high-fat diet produces hypomethylation of the Lep promoter in offspring adipose depots with increased leptin expression and circulating leptin, supporting that the maternal metabolic state can drive fetal epigenetic remodeling of leptin signaling pathways (24). Such persistent alterations in early leptin exposure may reprogram the hypothalamic energy homeostasis and thereby cause increased susceptibility to obesity and metabolic dysfunction later in life (25). These findings lend credibility to the hypothesis that maternal metabolic exposures in utero can epigenetically reprogram leptin signaling pathways and shape the offspring's susceptibility to obesity.

Adiponectin (ADIPOQ) is a key adipokine that enhances insulin sensitivity and promotes fatty acid oxidation. It is also increasingly being recognized to have a role in the developmental programming contributing to metabolic health. In gestational diabetes mellitus (GDM), ADIPOQ methylation showed a modest increase in maternal adipose tissue, but at the same time showed marked hypomethylation in the cord blood, which is strongly associated with a higher birth weight (26). The inverse association between ADIPOQ methylation and gene expression in adipose tissue reiterates the importance of epigenetic regulation in governing maternal and fetal insulin sensitivity, with potential long-term implications for metabolic growth trajectories (27). Sustained alterations in adiponectin signaling during critical developmental windows may influence adipocyte differentiation, lipid partitioning, and pancreatic β-cell function, thereby programming postnatal growth patterns and long-term metabolic susceptibility (28, 29).

The influence of GDM on fetal body composition has been further supported by the epigenetic modifications which were noted in the genes which are key regulators of adipogenesis. Placental methylation of bone morphogenetic protein 7 (BMP7) and PR/SET domain 16 (PRDM16) is reduced in pregnancies complicated by GDM, accompanied by increased expression of the PPARG coactivator 1 alpha (PPARGC1α). Cord-blood leptin levels are positively correlated with PRDM16 and PPARGC1α methylation, suggesting that these genes play a role in the epigenetic regulation of fetal adiposity (30). Rodent studies demonstrate that maternal metabolic perturbation alters DNA methylation and expression of Prdm16 and Ppargc1α in offspring adipose tissue, disrupting thermogenic and mitochondrial programming and contributing to altered adiposity, consistent with placental methylation findings in human GDM pregnancies (30, 31). Lipid metabolism pathways are similarly affected in GDM. The decreased placental methylation of lipoprotein lipase (LPL) is associated with increased gene expression. On the contrary, higher LPL methylation correlates positively with birth weight, pointing to a role for altered lipid handling in fetal growth programming (32).

Insulin-related pathways also exhibit epigenetic dysregulation, characterized by increased promoter methylation and reduced expression of glucose-6-phosphate dehydrogenase (G6PD), as well as hypermethylation of insulin-like growth factor binding proteins (IGFBPs) in the placenta of GDM. It has been shown that IGFBP-1, IGFBP-2, and IGFBP-6 in the placenta, but not in mixed blood, correlate with birth weight, reinforcing the tissue-specific roles of insulin-related epigenetic regulation in mediating fetal growth (33). IGFBPs function to regulate the half-life, receptor accessibility, and spatial distribution of IGFs. Epigenetic downregulation of IGFBPs may enhance free IGF concentrations, augmenting IGF receptor activation and downstream PI3K-Akt-mTOR signaling pathways that promote trophoblast proliferation, nutrient transport, and fetal growth (34).

Beyond protein-coding genes, maternally imprinted loci such as delta-like noncanonical notch ligand 1 (DLK1) and long noncoding RNA maternally expressed 3 (MEG3*)* are also affected by GDM. Hypermethylation of the DLK1 promoter reduces expression on both the maternal and fetal sides of the placenta, with methylation levels on the fetal side significantly associated with birth weight (35). Similarly, MEG3 (a tumor long noncoding RNA that regulates gene expression through interaction with chromatin-modifying complexes and modulation of the p53 and PI3 K/Akt pathways, (36)) promoter hypermethylation in GDM placentas results in decreased transcript levels, methylation being positively correlated with maternal fasting glucose and offspring birth weight (37). These findings suggest that maternal hyperglycemia alters the dynamics of imprinting, with functional consequences for the regulation of placental and fetal growth. In GDM pregnancies, reduced methylation of insulin-like growth factor 2 (IGF2) and increased methylation of maternally expressed H19 imprinted transcript (H19) have been reported, along with corresponding increases and decreases in gene expression, respectively, in placental tissue and cord blood (38). These imprinting alterations are suggested to be associated with variations in birth weight, suggesting that maternal glucose dysregulation can influence fetal growth trajectories through differential epigenetic regulation of growth factor pathways. Beyond growth factors, placental serotonergic signaling has also been implicated in this process. Elevated methylation of the serotonin transporter (SLC6A4) in GDM placentas correlates with increased gene expression, higher maternal plasma glucose, and elevated neonatal birth weight percentile. At the same time, it is inversely associated with head circumference (39). It has been suggested that maternal hyperglycemia disrupts placental serotonin regulation by altering tryptophan metabolism and inflammatory signaling. This imbalance could affect placental blood flow and nutrient transfer, influencing fetal growth. Because the placenta supplies serotonin to the developing fetal brain early in pregnancy, altered SLC6A4 expression may also impact neurodevelopment and head circumference (40).

Although candidate gene studies highlight specific pathways, genome-wide analyses emphasize the pervasive nature of epigenetic remodeling. Thousands of genes in the placenta and cord blood are differentially methylated between GDM and control pregnancies, and hundreds of genes show significant associations with birth weight (41). Candidate loci, such as rho guanine nucleotide exchange factor 11 (ARHGEF11), are hypomethylated in macrosomic infants of mothers with GDM, with methylation inversely correlated with maternal glucose levels and neonatal birth weight (42). ARHGEF11 activates rho GTPase signaling, which regulates cytoskeletal dynamics, cell proliferation, and insulin signaling pathways (43). However, the findings on global DNA methylation are inconsistent. Some studies report placental hypomethylation associated with a smaller newborn size (44), while others describe hypermethylation linked to large infants (45). These discrepancies reflect tissue-specific, context-dependent, or population-related differences in epigenetic responses to maternal hyperglycemia.

### Maternal pre-pregnancy BMI and gestational weight gain

Evidence suggests that maternal underweight and obesity before pregnancy can influence a newborn's DNA methylation patterns, which in turn affect body fat development later in life through intrauterine mechanisms. However, the increase in maternal weight during pregnancy has little effect on the methylation of the offspring's DNA. The nutritional status plays a more critical role in shaping the early epigenetic environment and possibly influencing long-term health outcomes (46). In a cross-sectional study of 114 full-term neonates, pre-pregnancy maternal BMI was associated with reduced methylation at 5 LEP CpG sites in cord blood, with lower methylation strongly associated with higher neonatal leptin, fat mass, and percentage body fat (47). Higher intrauterine leptin exposure caused by pre-pregnancy obesity might cause increased fetal adiposity and could influence the maturation of hypothalamic appetite-regulatory circuits, potentially predisposing offspring to altered energy balance and long-term obesity risk (48). These findings provide evidence that maternal BMI can epigenetically regulate LEP expression in utero, thus programming neonatal adiposity and increasing the risk of obesity. Parallel epigenetic signals have been detected in noninvasive tissues. Salivary DNA methylation analysis in preschool-aged children identified 17 CpG sites associated with maternal BMI, several of which are related to genes involved in obesity, insulin signaling, and circadian regulation. Pathway enrichment implicated homocysteine/methionine metabolism and cysteine biosynthesis processes central to one-carbon metabolism and methyl group availability, supporting the biological plausibility of maternal BMI-driven epigenetic programming (49).

Large-scale epigenome-wide association studies (EWAS) further support the role of maternal BMI in shaping offspring methylation. In one cohort of more than 1000 mother–child pairs, maternal obesity and underweight were associated with distinct, nonoverlapping methylation changes at 28 and 1621 CpG sites, respectively. Most of the alterations showed a positive correlation with the adiposity of the offspring. The potent effects of maternal vs paternal BMI suggest an intrauterine mechanism, while gestational weight gain has a limited influence (46). Similarly, analysis of cord-blood methylation in 308 Black mother–infant pairs identified 20 CpG sites associated with maternal BMI, including loci in genes involved in inflammatory signaling, angiopoietin like 2 (ANGPTL2), lipid metabolism, adenylate cyclase 3 (ADCY3), and cardiovascular disease pathways, Erb-B2 receptor tyrosine kinase 2 (ERBB2), suggesting that maternal obesity may predispose offspring to cardiometabolic disease through epigenetic modification (50). Evidence from animal models supports these findings, as in mice, maternal high-fat diet exposure induces widespread DNA methylation changes in the offspring's liver, with enrichment in inflammatory and metabolic pathways. This is shown to be accompanied by increased adiposity and metabolic dysfunction in adulthood, reinforcing the concept that maternal obesity can remodel the offspring methylome in metabolically active tissues, causing cardiometabolic risk (51).

Meta-analyses offer a broader perspective on these associations. The Pregnancy and Childhood Epigenetics (PACE) consortium evaluated more than 9000 mother–newborn pairs and identified widespread, although modest, methylation differences associated with maternal BMI at 9044 CpG sites. After stringent adjustment for cell-type composition, it has been shown that 104 CpG sites remained significantly associated, of which 72 demonstrated continued association into adolescence. However, causal inference analyses suggested that the actual intrauterine effects could be confined only to a relatively small subset of these loci. Many of the observed associations may reflect shared genetic architecture or postnatal environmental exposures, underscoring the challenge of unraveling intrauterine programming from confounding influences in epigenetic analysis (52).

Sex-based analyses further reveal that the epigenetic effects of maternal BMI may show substantial differences between male and female offspring. The Newborn Epigenetics Study (NEST) has shown that maternal obesity was associated with differential methylation at 876 CpG sites in the female newborns, while differential methylation was seen only at 293 in males, with minimal overlap between the 2 sets. The antigen processing gene, TAP binding protein (TAPBP), has shown consistent changes in the CpGs in the female offspring, and several of these loci identified were correlated with BMI and blood pressure at 4 to 5 years of age. The fact that sex-specific patterns exist suggests that maternal BMI can differentially influence cardiometabolic trajectories depending on the sex of the offspring, although replication in independent cohorts remains limited (53).

Recent genome-wide analyses have reinforced the notion that maternal BMI exerts epigenetic effects on multiple tissues. In the Gen3G cohort, paired cord blood and placental samples from 437 newborns revealed consistent methylation at CpGs within corticotropin-releasing hormone binding protein (CRHBP) and the coiled coil domain containing 97 (CCDC97) genes, suggesting possible programming of the hypothalamic-pituitary-adrenal axis and stress regulation (54). CRHBP regulates the bioavailability of corticotropin-releasing hormone (CRH) and thereby modulating activation of the Hypothalamic–Pituitary–Adrenal (HPA) axis. Altered methylation of CRHBP may influence its expression and change the free CRH levels in the placenta and fetal circulation, which could influence HPA axis set-point calibration, stress reactivity, and metabolic regulation later in life (55). Similarly, analysis of the Boston Birth Cohort identified hundreds of cord-blood CpGs associated with maternal BMI, of which 123 were also associated with childhood overweight or obesity between ages 1 and 18. Mediation models implicated 14 CpGs as possible mechanistic links between maternal BMI and offspring obesity outcomes, providing evidence that epigenetic marks at birth may serve as mediators of the risk of intergenerational obesity (56).

Placental epigenetic signatures offer additional information on the mechanisms of fetal growth programming. Studies within the ELGAN cohort and others have shown that methylation at the mesoderm-specific transcript (MEST), histone deacetylase 4 (HDAC4), and nuclear receptor subfamily 3 group C member 1 (NR3C1) is associated with growth trajectories and obesity risk by age 10, underscoring the placenta as both a nutrient-sensing and epigenetically active organ that contributes to long-term health programming (57). In rats, maternal protein restriction induces persistent DNA methylation changes in the offspring's liver at metabolic gene promoters, resulting in long-term alterations in gene expression and metabolic phenotype (58).

Although there is compelling mechanistic evidence from targeted human studies and studies using experimental animal models, large-scale population data reveal a complex landscape. A meta-analysis by the PACE consortium, involving nearly 10 000 mother–child pairs in 19 cohorts, identified widespread associations between maternal pre-pregnancy BMI and offspring DNA methylation. However, after rigorous adjustment for estimated blood cell type composition and replication in adolescent samples, only 8 CpG sites remained robustly associated, mapping to or near Sp6 transcription factor (SP6), N-terminal EF-hand calcium binding protein 3 (NECAB3), agrin (AGRN), secreted frizzled related protein 1 (SFRP1), glucosidase, beta, acid pseudogene 1 (GBAP1), long intergenic non–protein-coding RNA 1622 (LINC01622), urocortin (UCN), and serine protease 21 (PRSS21) (52), although the effect sizes were small (<0.2% per BMI unit). Together, they suggest that maternal BMI may subtly influence transcriptional regulation and signaling systems that coordinate placental development, adipogenesis, and neuroendocrine calibration (59-61).

In the NEST, paternal preconception obesity was inversely associated with offspring IGF2 DMR methylation in cord blood, regardless of maternal BMI or newborn characteristics, suggesting that epigenetic alterations acquired during spermatogenesis can be transmitted to offspring (62). Complementary evidence from a larger genome-wide study demonstrated that paternal BMI was associated with specific changes in DNA methylation at birth, including hypomethylation at the regulatory sites of the transcription factor AP-2 gamma (TFAP2C)/bone morphogenetic protein 7 (BMP7) and ARF GTPase activating protein 3 (ARFGAP3), with the latter being linked to lower birth weight and higher BMI score by age three. Some of these paternal BMI-associated methylation marks persisted into early childhood, providing strong evidence for paternal contributions to the intergenerational risk of obesity (63).

Beyond classical metabolic regulators such as LEP and ADIPOQ, epigenetic modifications at the hypoxia-inducible factor 3 alpha (HIF3A) locus have attracted considerable interest due to their associations with adiposity throughout the life course. HIF3A is a transcription factor central to cellular adaptation to hypoxia and plays a key role in the remodeling of adipose tissue during fat expansion. Epigenetic regulation of HIF3A has emerged as one of the most reproducible associations with adiposity. Previous studies involving multiple cohorts and age groups have shown that hypermethylation within intron 1 of HIF3A has been consistently associated with increased BMI, increased body fat percentage, and insulin resistance (64). Mechanistically, such methylation may weaken the adaptive response of adipose tissue to hypoxia during fat accumulation, thus impairing metabolic dysfunction. The fact that these associations could be reproduced in some of the later studies led to the proposal that HIF3A methylation could serve as an early biomarker of obesity and cardiometabolic risk, suggesting that it influences methylation levels and is involved in a genetic-epigenetic interplay in childhood obesity susceptibility.

Furthermore, in the Barwon Infant Study, cord-blood methylation from 938 infants was assessed in 2 HIF3A regions. Although methylation levels in the widely studied region did not show any association with the birth weight, adiposity, or GDM, a second region exhibited more complex patterns, where methylation was decreased with male infants and preeclampsia but increased with GDM and gestational age. The strong genetic influences at this locus underline the importance of considering both genetic and environmental contributions to early-life epigenetic variations. These observations also suggest that pregnancy complications, including preeclampsia and maternal glycemia, can shape the infant epigenome at loci relevant to vascular and metabolic function, potentially also influencing long-term cardiometabolic risks (65).

Although the cross-sectional associations have been established, the directionality of the HIF3A adiposity relationship is still not well understood. In the Avon Longitudinal Study of Parents and Children (ALSPAC), a longitudinal analysis of nearly 1000 pairs of parents and children underwent an integrated methylation, adiposity, and genetic analysis to evaluate causality. The findings suggested that higher BMI appears to influence methylation levels, and HIF3A methylation is unlikely to be a primary driver of adiposity (66). Although Mendelian randomization analyses supported this reverse causality, the availability of only limited statistical power prevented making definitive conclusions. The study also identified an intergenerational effect of maternal BMI on the methylation of the offspring in HIF3A, raising the possibility that shared metabolic environments may confound the variations observed. These findings position HIF3A methylation as a strong epigenetic correlate of adiposity rather than a confirmed causal mediator. This evidence also underscores the broader challenge within epigenetic epidemiology of separating cause from consequence effects. It suggests that HIF3A methylation may function more reliably as a biomarker of adiposity and metabolic stress than as an initiating factor in the development of childhood obesity. Future mechanistic studies, including clustered regularly interspaced short palindromic repeats (CRISPR)–based methylation editing and high-resolution adipose tissue profiling, will be essential to clarify whether HIF3A represents a viable target for intervention or a marker of cumulative metabolic burden.

Early gestational weight gain was associated with altered methylation at CpG sites within the matrix metallopeptidase 7 (MMP7), potassium 2-pore domain channel subfamily K member 4 (KCNK4), transient receptor potential cation channel subfamily M member 5 (TRPM5), and nuclear factor kappa B subunit 1 (NFKB1) genes, which are involved in immune signaling and cellular homeostasis (67). In a larger ALSPAC study, maternal obesity was associated with 28 differentially methylated CpG, including succinate-CoA ligase GDP-forming subunit Beta (SUCLG2), FAM129B intron CAGE-defined low expression enhancer (FAM129B), and kinesin family member 15 (KIF15). Differential methylation at loci including SUCLG2, FAM129B, and KIF15 suggests that maternal obesity may influence offspring epigenetic regulation of mitochondrial energy metabolism, stress-responsive signaling, and cell-cycle control, supporting a model of distributed metabolic and proliferative reprogramming rather than single-gene effects (68-70). In contrast, maternal underweight was associated with 1621 sites, including testes expressed 14, intercellular bridge forming factor (TEX14), zinc finger protein 783 (ZNF783), and mevalonate diphosphate decarboxylase (MVD), with negligible overlap (46). Thus, maternal underweight was linked to widespread methylation differences enriched in genes involved in sterol biosynthesis (MVD), cell division (TEX14), and transcriptional regulation (ZNF783), suggesting a broader epigenetic adaptation to nutrient constraint that may influence lipid metabolism, cellular proliferation, and developmental growth trajectories. The strongest associations observed between maternal and paternal BMI emphasize the contribution of the intrauterine environment to epigenetic programming. Maternal obesity has been associated with widespread epigenetic changes in the tissues of offspring, many of which correspond to pathways that regulate adipogenesis, lipid metabolism, and immune function. Hypomethylation of adipogenic transcriptional regulators, including CCAAT enhancer binding protein Beta (C/EBP-β) and the zinc finger protein genes, has been observed in the progeny of obese mothers, which could enhance adipocyte differentiation and predispose to later adiposity (31, 71).

In contrast, hypermethylation of fatty acid oxidation genes (protein kinase AMP-activated noncatalytic subunit Gamma 2 [PRKAG2], acetyl-CoA carboxylase Beta [ACACB], carnitine palmitoyltransferase 1A [CPT1A], succinate dehydrogenase complex subunit C [SDHC]) was detected in mesenchymal stem cells from cord blood of neonates born to obese mothers, with positive associations with neonatal adiposity (72). Hypermethylation of key fatty acid oxidation genes in cord-blood mesenchymal stem cells suggests reduced oxidative capacity and a potential shift toward lipid storage and adipogenic differentiation, which may contribute to increased neonatal adiposity. In the context of maternal obesity-associated inflammation, such epigenetic modification may reflect altered immune programming, with potential long-term consequences for inflammatory regulation in the offspring (73). Similarly, elevated methylation of aryl hydrocarbon receptor repressor (AHRR), an inhibitor of peroxisome proliferator-activated receptor Gamma (PPARγ) driven adipogenesis, further implicates epigenetic dysregulation of adipocyte development in maternal obesity (74, 75). Collectively, these studies demonstrate that maternal obesity imprints durable epigenetic alterations in multiple biological systems, predisposing offspring to both metabolic dysfunction and altered immune competence.

Significantly, lifestyle interventions during pregnancy may attenuate obesity and epigenetic modifications in offspring. In the UPBEAT randomized controlled trial of 557 obese pregnant women, maternal GDM and post-load glucose levels were associated with widespread differences in offspring methylation in cord blood, which included 242 CpG for GDM, and hundreds more for fasting and 1-hour glucose. The highest site mapped to Leucine Rich Repeat Containing G protein-coupled receptor 6 (LGR6). However, in the intervention arm (diet and physical activity), the number and effect size of these associations were markedly reduced, suggesting that the intrauterine epigenetic programming of obesity risk is at least partially modifiable during pregnancy (76). Parallel evidence from animal models supports the flexibility of intrauterine epigenetic programming to environmental cues. In mice, maternal exercise prevented high-fat diet–induced hypermethylation of the Ppargc1α promoter in offspring skeletal muscle and restored metabolic function (77).

The observed associations between maternal BMI and methylation of the offspring are consistent with fundamental principles of developmental epigenetics. During early embryogenesis, global erasure and reestablishment of DNA methylation patterns create a window of heightened plasticity, during which environmental inputs, including maternal nutrition and metabolic status, can durably alter the epigenome. Although it has been proposed that DNA methylation patterns are mitotically heritable, they are modifiable during key developmental windows, including gestation, infancy, puberty, and aging, where environmental inputs can exert a disproportionate influence, altering long-term physiology (78). Therefore, maternal obesity operates during the sensitive phase of epigenome establishment, with consequences which can be lasting long enough to exert altered metabolic regulation in the offspring.

Mechanistic studies carried out in model systems and targeted human cohorts have supported the possibility that prenatal exposures can induce long-lasting methylation changes at regulatory loci such as proopiomelanocortin (POMC) and LEP, with functional consequences on energy balance and adiposity which persist well into adulthood (79). On the other hand, large-scale EWAS studies often identify numerous associations which are of modest magnitude, many of which weaken after adjustment for cell composition or fail to replicate across different populations. This discrepancy suggests that while some of the epigenetic marks may serve as bona fide mediators of developmental programming, others likely reflect the effects of population heterogeneity, shared genetic architecture, or momentary environmental impact. Distinguishing actual epigenetic programming from correlation remains a central challenge, which needs integrative approaches that combine genetic, functional, and longitudinal analysis.

### Maternal nutrition and lifestyle

Maternal nutrition is one of the powerful modifiers of the fetal DNA methylation marks. Even in nutrient-replete populations, dietary variations can induce widespread changes in methylation, although it is true that only a subset of these translate into gene-specific functional changes (80).

The effect of nutrition can be seen throughout the reproductive continuum, from preconception to gestation and lactation. Variations in intake of macronutrients such as protein, fat, fructose, and micronutrients such as vitamin D, folate, choline, and other methyl donors shape DNA methylation in genes governing metabolism, appetite, and growth. These epigenetic changes have been linked to a range of outcomes in the offspring, including dysregulated glucose and lipid metabolism, altered adipokine profiles, hypertension, fatty liver disease, and increased susceptibility to obesity, all from childhood (81). The consistency which has been seen in these associations in dietary exposures highlights the remarkable sensitivity of the developing epigenome, including that of DNA methylation, to nutritional environments during periods of increased plasticity.

Long-term cohort studies provide compelling evidence that the effects of maternal malnutrition are durable and can leave enduring epigenetic signatures that bridge prenatal exposures with those of the adult cardiometabolic risks. In the Motherwell cohort, maternal consumption of highly unbalanced diets during pregnancy was associated with persistent differences in methylation at the regulatory regions of hydroxysteroid 11-beta dehydrogenase 2 (HSD2), glucocorticoid receptor (GR), and IGF2 regulatory regions. These methylation differences were correlated with the neonatal body measures and later adult adiposity, blood pressure, and cortisol levels (81). Specifically, it has been shown that the elevated methylation at GR exon 1F was the most pronounced among individuals with the greatest prenatal exposure to dietary imbalance. Differential methylation at HSD2, GR exon 1F, and IGF2 suggests coordinated reprogramming of glucocorticoid signaling and growth pathways (82, 83). Together, these provide a mechanistic framework linking prenatal dietary imbalance to long-term alterations in stress physiology, adiposity, and blood pressure (84). These findings illustrate how adverse early developmental environments become biologically embedded through epigenetic changes, shaping cardiovascular and metabolic growth trajectories throughout life.

Metastable epiallele regions at which methylation is established stochastically during early embryogenesis are susceptible to maternal nutritional conditions and may contribute to individual differences in disease susceptibility (85, 86). Several such loci have demonstrated predictive potential; for example, placental methylation at MEST and HDAC4 genes in the ELGAN cohort has been shown to be associated with childhood obesity risk up to age ten (87-89).

Historical famines have provided natural experiments that illustrate the lasting imprint of early nutritional adversity. Babies exposed to the Dutch Hunger Winter (1944-1945) or the Chinese famine (1959-1961) in utero show higher rates of adult obesity, glucose intolerance, and cardiovascular disease compared to their unexposed peers (90, 91). Epigenetic analyses have revealed that there is persistent hypomethylation of gene IGF2 among those exposed around conception, and these changes remain detectable even 6 decades later (92). This seminal observation has demonstrated that the epigenome could retain a molecular memory of early nutritional stress, particularly during the period when methylation patterns undergo widespread reestablishment in the embryo. Subsequent studies have identified additional exposure-related methylation differences in genes that are involved in metabolism and immune function, including insulin (INS), IGF2, GNAS antisense RNA 1 (*GNAS-AS1*), MEG3, interleukin 10 (IL-10), and LEP (93). These findings suggest that adverse prenatal nutrition reprograms multiple metabolic and inflammatory pathways. The timing of exposure, especially around the time of conception, further emphasizes the increased vulnerability of the early developmental epigenome, consistent with the hypothesis of developmental origins of health and disease (DOHaD). Parallel evidence from animal models supports the persistence of early nutritional epigenetic programming. In mice, in utero undernutrition induced lasting DNA methylation alterations in metabolic tissues such as the liver and germ cells, accompanied by impaired glucose tolerance and intergenerational metabolic dysfunction (94), reinforcing the concept that early embryonic nutritional stress can durably reprogram the metabolic epigenome.

Beyond exposure to famine, a wide range of maternal nutritional environments, including overnutrition, high-fat diets, and micronutrient imbalance, have been associated with methylation changes in genes central to appetite regulation (LEP), lipid metabolism (PPARγ), and energy homeostasis (fat mass and obesity-associated protein [FTO]). Modifications in these critical genes can predispose the offspring to increased adiposity and metabolic dysfunction, supporting the DOHaD paradigm (95, 96). In some cases, these modifications persist over time, acting as an “epigenetic memory” that maintains the influence of early exposures well into adulthood. This persistence provides a plausible mechanistic explanation for the intergenerational transmission of obesity and the risk of metabolic disease.

Together, evidence from global methylation studies, birthweight-associated CpG, imprinted loci, and famine cohorts converges on a model in which the intrauterine environment calibrates epigenetic regulation of growth and metabolism. Although some methylation differences may be transient reflections of fetal growth states, others, particularly at imprinted and metabolic genes, persist into later life and contribute to the increased risk of metabolic diseases, including obesity (97-99). This interplay of nutrition, growth, and epigenetics highlights the importance of the periconceptional and gestational environments in shaping lifelong metabolic health.

**Figure 3:**
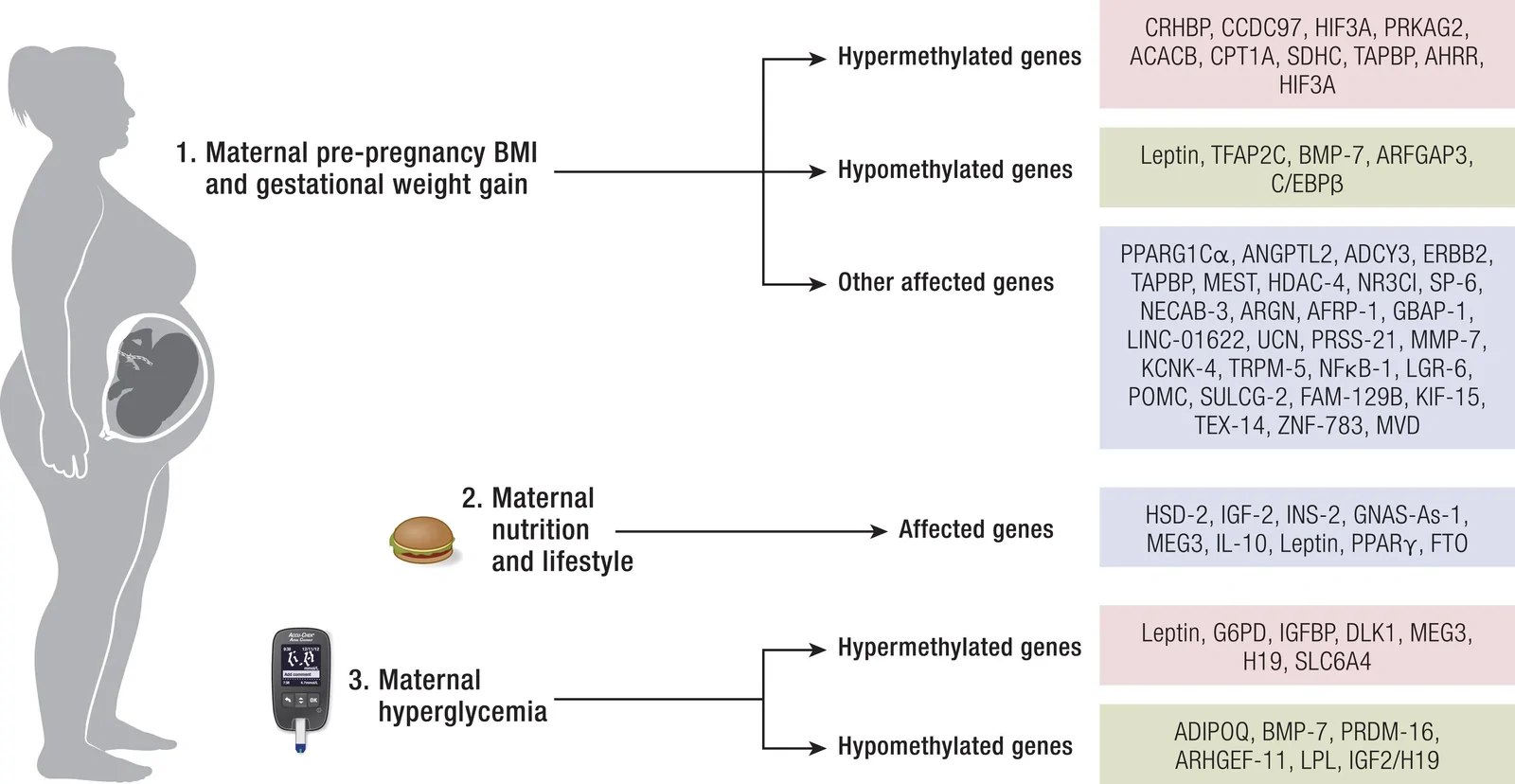
Prenatal environmental factors and DNA methylation signatures associated with offspring adiposity. Summary of maternal risk factors and the offspring genes they epigenetically influence through DNA methylation. Genes are grouped according to methylation status (hypermethylated, hypomethylated, or with unresolved/variable directionality), as indicated by the labels adjacent to each gene list.

The prenatal environmental factors and DNA methylation signatures associated with offspring adiposity are summarized in Fig. 3 and Table 1.

**Table 1:** Described DNA methylation signatures of childhood obesity

Environmental factor	Methylation change	Pathway affected	Tissue	Reference
**Prenatal environmental factors and DNA methylation signatures of adiposity in offspring**				
Obesity	Hypermethylation at LEP	Appetite regulation	Blood	(22)
Gestational diabetes mellitus	Hypomethylation at ADIPOQ	Insulin signaling, metabolism	Cord blood	(27)
Gestational diabetes mellitus	Hypomethylation at BMP7, PRDM 16	Thermogenic and mitochondrial programming	Placenta, adipose tissue	(30, 31)
Gestational diabetes mellitus	Hypomethylation at LPL	Lipid metabolism	Placenta	(32)
Gestational diabetes mellitus	Hypermethylation at G6PD, IGFBP	Metabolism	Placenta	(33)
Gestational diabetes mellitus	Hypermethylation at MEG3, DLK1	Growth	Placenta	(35, 37)
Gestational diabetes mellitus	Hypomethylation at IGF2/H19	Insulin signaling	Placenta, cord blood	(38)
Gestational diabetes mellitus	Hypermethylation at SLC6A4	Appetite regulation, metabolism	Placenta	(39, 40)
Gestational diabetes mellitus	Hypomethylation at ARHGEF11	Cytoskeleton	Placenta	(44, 45)
Obesity	Hypomethylation at LEP	Appetite regulation	Cord blood	(47)
Obesity	Differential methylation at ANGPTL2, ADCY3, ERBB2	Cardio-vascular development	Cord blood	(50)
Obesity	Differential methylation at TAPBP	Metabolism	Cord blood	(53)
Obesity	Differential methylation at CRHBP, CCDC97	Hypothalamic-pituitary-adrenal axis	Cord blood	(54)
Obesity	Hypermethylation at MEST, HDAC4, NR3C1	Metabolism	Placenta	(57)
Obesity	Differential methylation at SP6, NECAB3, AGRN, SFRP1, GBAP1, LINC01622, UCN, PRSS21	Placental development, adipogenesis, neuroendocrine development	Blood	(52)
Obesity	Hypomethylation at TFAP2C, BMP7, ARFGAP3	Metabolism, development	Blood	(63)
Obesity, body fat composition, insulin resistance	HIF3A hypermethylation	Lipid metabolism	Blood	(64)
Gestational diabetes mellitus, gestational age	HIF3A hypermethylation	Metabolism, cardio-vascular development	Umbilical cord-blood mononuclear cells	(65)
Preeclampsia	HIF3A hypomethylation	Metabolism, cardio-vascular development	Umbilical cord-blood mononuclear cells	(65)
Early gestational weight gain	Differential methylations at MMP7, KCNK4, TRPM5, NFkB	Immune signaling, cellular homeostasis	Cord blood	(67)
Maternal obesity	Differential methylations at SUCLG2, FAM129B, KIF15	Metabolism, cell proliferation	Multiple tissues and HeLa cells	(68-70)
Maternal underweight	Differential methylations at TEX14, ZNF783, MVD	Lipid metabolism, cell proliferation	Blood	(46)
Maternal obesity	Hypomethylation at C/EBP-β, ZNF proteins	Lipid metabolism	White adipose tissue	(31, 71)
Maternal obesity	Hypermethylation at PRKAG2, ACACB, CPT1A, SDHC	Fatty acid oxidation	Mesenchymal stem cells	(72)
Maternal obesity	Hypermethylation at TAPBP	Chronic inflammation	Cord blood	(53)
Maternal obesity	Hypermethylation at AHRR	Lipid metabolism	Cervical swab, blood cells	(74, 75)
Maternal nutritional imbalance	Differential methylation at HSD2, GR, IGF2	Glucocorticoid signaling, growth pathways	Multiple tissues	(81)
Maternal nutritional imbalance	Differential methylation at INS, IGF2, GNAS-AS1, MEG3, IL-10, LEP	Metabolism, inflammatory pathways	Blood	(93)
Maternal nutritional imbalance	Differential methylation at LEP, Pparγ, FTO	Metabolic pathways	Blood	(95, 96)
**Perinatal environmental factors and DNA methylation signatures of adiposity in offspring**				
Early gestational carbohydrate intake	Differential methylation at Rxrα	Lipid metabolism, insulin sensitivity	Umbilical cord tissue	(100)
Obesity	Hypermethylation at HIF3A	Lipid metabolism	Umbilical cord	(101)
Parental overnutrition	Hypomethylation at LEP, hypermethylation at POMC	Regulation of metabolism, energy balance	Placenta	(102, 103)
**Postnatal environmental factors and DNA methylation signatures of adiposity in offspring**				
Neonatal birth weight	Differential methylation in ARID5B, XRCC3	DNA repair pathways	Cord blood	(104)
Neonatal birth weight	Hypomethylation at the DMR region at IGF2	Insulin signaling	Cord blood	(105-110)
Neonatal birth weight	Hypermethylation at TYRO3, SMYD3, ZNF117, MTF2, SETBP1, SPG21, hypomethylation at DBH, ARHGAP17, PPP2R5C	Metabolic pathways	Blood	(111)
Gestational diabetes mellitus	Hypomethylation at Cdkn2a, Cdkn2b	Cell cycle	Pancreatic islets	(112)
Dietary glycemic load, nutritional quality	Differential methylation at WDR27, CSF3	Insulin signaling, fat metabolism	Blood	(113)
Breastfeeding duration, parental socioeconomic status	Hypermethylation at LEP	Regulation of metabolism, energy balance	Blood	(114)
Neonatal adiposity	Differential methylation at HIF3A	Lipid metabolism	Blood	(115)
Childhood adiposity	Hypermethylation at POMC	Energy balance, appetite	Blood	(116)
Multiple factors	Differential methylations at VTRNA2-1, KPRP, PRLHR, MYLK2, MMP25, GFPT2	Metabolism	Cord blood	(95, 117)
Obesity, fat mass, fat-free mass	Differential methylations at SNED1, KLHL6, CFLAR, PRDM14, SOS1, ST6GAL1	Glucose and lipid metabolism	Blood	(118)
Multiple factors	Methylations at PEG10, MEST	Placental development, adipogenesis	Blood	(119)
Obesity	Differential methylations at SREBF1, ANRIL	Lipid metabolism	Blood	(120-122)
Maternal lifestyle (exercise, diet)	Methylation changes at DISC1, HERC2	Intracellular scaffolding, cytoskeletal organization, ubiquitin-mediated protein turnover	Blood	(123, 124)
Maternal exercise	Methylation changes at Ppargc1α	Insulin signaling	Skeletal muscle	(77, 125)
Gestational diabetes mellitus	Methylation changes at Igf2/H19	Insulin signaling	Liver, pancreas	(126)
Early-life methylation status	Methylation changes at SIM1	Appetite control	Blood	(127)
Early-life methylation status	Hypermethylations at UBASH3A, TRIM3	Immune regulation	Blood	(128)
Early-life methylation status	Hypermethylation at Pparγ	Lipid metabolism	Blood	(129)
Early-life methylation status	Differential methylation at ANRIL	Lipid metabolism	Adipose tissue	(130)
Early-life methylation status	Differential methylation at HNF4a	Lipid metabolism	Pancreas	(131)
Early-life methylation status	Hypermethylation at Pparγ	Lipid metabolism	Adipose tissue	(58, 132)
Early-life methylation status	Differential methylations at DNAJA4, TFR2, SMAD3, PLAG1, FGF1, HNF4A	Growth, energy metabolism	Cord blood	(133)
Early-life methylation status	Differential methylation at TACSTD2	Growth	Blood	(134)
Early-life methylation status	Hypermethylation at POMC	Appetite, energy balance	Blood	(135)
Early-life methylation status	Hypomethylation at IGF2, hypermethylation at CYP27B1	Growth factor, vitamin D pathways	Blood	(136)
Early-life methylation status	Differential methylation at ZAC1	Growth regulation and endocrine signaling	Blood	(137)
Early-life methylation status	Hypermethylation at RAX2, hypomethylation at APOA5, CES1	Lipid metabolism	Blood	(138)
Early-life methylation status	Differential methylations at PTPRS, VIPR2, PER3	Metabolism and energy regulation	Blood, placenta	(139, 140)
Early-life methylation status	Hypomethylation at LEP	Metabolism and energy regulation	Saliva	(141)
Obesity, insulin resistance	Hypomethylation at LEP, ADIPOQ	Metabolism and energy regulation	Blood	(142)
Subcutaneous adiposity, lipid profile	Hypermethylation at IGF2	Insulin signaling	Blood	(143)
Obesity	Hypermethylation at UBASH3A, hypomethylation at TRIM3	Immunoregulation	Blood	(144)
Obesity	Differential methylation at FZD7, PRLHR, EIF6	Growth regulation	Blood	(145)
Adiposity, insulin resistance	Hypermethylation at LY86	Inflammation	Blood	(146)

### Perinatal environmental determinants and DNA methylation signatures of offspring adiposity

Environmental factors during the perinatal period can shape DNA methylation patterns in the newborn, with implications for later health. Such modifications may mediate the DOHaD by altering gene expression programs linked to metabolism, cardiovascular function, and growth, thus increasing risks of conditions such as hypertension, insulin resistance, and cardiovascular disease in later life (147).

### Maternal nutrition and obesity

Candidate gene approaches have shown that DNA methylation at specific loci in umbilical cord blood can predict the results of adiposity years later. Using Sequenom MassARRAY, methylation at retinoid X receptor alpha (RXRα) and nitric oxide synthase 3 (ENOS) was shown to explain more than 25% of the variance in childhood fat mass and percentage body fat at 9 years of age, together with sex. RXRα methylation was inversely related to maternal carbohydrate intake in early pregnancy, highlighting the nutritional sensitivity of this locus. In a replication cohort, associations with RXRα persisted, while ENOS did not (100). Methylation at RXRα, a central nuclear receptor coordinating PPAR-dependent lipid metabolism and adipogenesis, has been suggested to provide a mechanistic link between early maternal diet and long-term adiposity (148). These findings suggest that prenatal methylation in metabolically relevant genes may serve as predictive biomarkers of obesity risk, with RXRα reflecting maternal nutritional exposures.

The availability of methyl donors, such as folate-related metabolites, is another important determinant of fetal DNA methylation. A genome-wide analysis of the cord blood from 12 newborns has shown that the CpG methylation patterns were strongly correlated with plasma homocysteine, global LINE-1 retrotransposable element ORF1 protein-like (*LINE-1*) methylation, and birthweight centile. CpGs associated with birth weight or *LINE-1* were disproportionately enriched within CpG islands, suggesting that folate-related metabolites can influence epigenetic regulation in transcriptionally relevant regions (101). These findings are consistent with epidemiological evidence indicating that both periconceptional and late gestational folate status can shape outcomes of the pregnancies through epigenetic programming.

There is also evidence which suggests that HIF3A methylation patterns are already detectable at birth. In a cohort study of 991 infants, increased methylation at three CpG sites within intron 1 of HIF3A, measured in the umbilical cord DNA, was shown to be positively associated with the newborn and infant adiposity (149). Although the effect sizes were smaller than those observed in adults, the presence of this association already at birth indicates that early developmental environments contribute to changes in the HIF3A methylation, resulting in epigenetic variation. However, although strong genetic influences on methylation levels were evident, this could not be linked to individual prenatal exposure, underscoring the multifactorial nature of early-life epigenetic regulation.

Epigenetic changes at transcription factor binding sites further illustrate the functional consequences of altered DNA methylation. For example, methylation at a GATA binding protein 1 (GATA1) binding site within the carboxypeptidase A3 (CPA3) promoter changes its expression in the adipose tissue, with potential consequences for adiposity (130). Similar to this, methylation at the CDKN2B antisense RNA 1 (ANRIL) locus, which is located near cyclin-dependent kinase inhibitor 2A (CDKN2A), interrupts estrogen receptor-α binding and has been shown to be associated with adiposity at 6 years of age (102). These examples demonstrate that DNA methylation marks can act not only as biomarkers but as functional modifiers of transcriptional regulation at key metabolic loci. Among the candidate genes, LEP and POMC stand out as central regulators of energy balance whose methylation patterns are susceptible to parental obesity and perinatal exposures. In a study that examined LEP methylation in maternal, cord blood, and placental tissues, maternal obesity was associated with reduced methylation in maternal blood. On the contrary, cord-blood methylation was higher in small-for-gestational-age infants, but lower in offspring of obese mothers. The genome (rs2167270) and infant sex further modulated methylation, suggesting multilayered regulation of leptin expression across tissues (103). The hypothalamic gene POMC, which is critical for the signaling of satiety, has been implicated as a key epigenetic target of overnutrition. In rodent models, neonatal overfeeding induced POMC promoter hypermethylation, impairing response to leptin and insulin and producing a metabolic syndrome phenotype (116). Translational evidence in humans confirmed that POMC promoter methylation in postmortem hypothalamic neurons strongly correlates with BMI, with marks established early in embryogenesis and like paternal methylation profiles, suggesting an intergenerational epigenetic mechanism (150). Effects of perinatal DNA methylation changes on offspring adiposity have been summarized in Table 1.

### Factors affecting postnatal growth trajectories and associated DNA methylation patterns

Postnatal DNA methylation contributes to the continued epigenetic remodeling that supports tissue maturation and functional development and differentiation after birth. It has been suggested that methylation patterns remain dynamic throughout early life, particularly within enhancer-like regulatory regions, which are present in the key organs such as the liver, heart, lung, and hippocampus. Some of these developmental changes are driven by the activity of Tet methylcytosine dioxygenases, TET2 and TET3, which are the key facilitators of active demethylation in response to metabolic and hormonal signals, including insulin signaling. Disruption of these pathways can affect normal patterns of demethylation and gene activation, which in turn can affect organ maturation. Environmental factors during the postnatal development, such as nutrition, physical activity, and hormonal fluctuations, can further alter DNA methylation and may influence long-term physiological function and increase susceptibility to diseases, including obesity (151).

### Birth weight

Birth weight is a well-established predictor of later health, with low and high extremes associated with an increased risk of cardiometabolic disease. Epigenetic regulation has been proposed as a mechanistic link between early growth trajectories and adult metabolic outcomes. Global DNA methylation, often assessed through *LINE-1* elements, acts as a proxy for genome-wide methylation status and chromosomal stability, which provide a view of these processes. In a cohort of 319 pairs of mothers and children, infants with low or high birthweight, as well as those born prematurely, exhibited lower *LINE-1* methylation in cord blood compared to infants appropriate for gestational age, even after accounting for maternal BMI and gestational weight gain (104). Although modest in magnitude, these reductions in global methylation suggest that epigenetic dysregulation accompanies both growth restriction and overgrowth at birth, potentially contributing to long-term susceptibility to cardiometabolic disease.

Genome-wide analyses have extended these observations to locus-specific associations. In the Norwegian MoBa cohort, epigenome-wide methylation profiling in 1046 infants identified 19 CpG sites associated with birth weight, including genes such as AT-rich interaction domain 5B (ARID5B) (associated with lower birth weight) and X-ray repair cross complementing 3 (XRCC3) (associated with higher birth weight), which implicates developmental and DNA repair pathways in fetal growth regulation (152). Similarly, a large meta-analysis of EWAS of the PACE consortium, involving 8825 neonates in 24 cohorts, identified 914 CpG sites associated with birth weight, corresponding to differences of ±178 g per 10% change in methylation (111). However, follow-up analyses revealed that <1.3% of these methylation differences persisted throughout childhood or adolescence and none into adulthood, suggesting that most associations may reflect transient responses to intrauterine growth rather than stable epigenetic programming. CpGs overlapped with sites previously linked to maternal smoking and BMI, but not with folate, indicating shared maternal metabolic and environmental pathways that shape the neonatal epigenome. Epigenome-wide analysis further suggests that birth weight modifies obesity-associated DNA methylation patterns in childhood. In a study of 16 children stratified by birth weight and obesity status, 521 differentially methylated regions (DMRs) were suggested to be associated with childhood obesity, of which 38 were also linked to birth weight. Both hypermethylation and hypomethylation were observed. Specific CpG sites showed hypomethylation in dopamine beta-hydroxylase (DBH), rho GTPase activating protein 17 (ARHGAP17), and protein phosphatase 2 regulatory subunit B″gamma (PPP2R5C) and hypermethylation in TYRO3 protein tyrosine kinase (TYRO3), SET and MYND domain containing 3 (SMYD3), zinc finger protein 117 (ZNF117), metal response element binding transcription factor 2 (MTF2), SET binding protein 1 (SETBP1), and SPG21 abhydrolase domain containing, maspardin (SPG21). Notably, differential methylation in DBH, TYRO3, and SMYD3 distinguished obese children born with low birth weight from healthy controls, supporting developmental contributions to obesity risk (105). Locus-specific studies of imprinted regions provide additional mechanistic insight. DNA methylation at the imprinted maternally expressed transcript locus IGF2/H19 (H19) has been a particular focus given its central role in fetal growth. In a Dutch cohort, small babies for gestational age had lower IGF2 DMR methylation in cord blood, while methylation in H19 and methylenetetrahydrofolate reductase (MTHFR) showed no association (106). It has been demonstrated that reduced IGF2 methylation can be correlated with greater postnatal weight gain at 3 months, highlighting its role in contributing to early growth trajectories. Genetic variation at the IGF2/H19 locus was associated with methylation changes, indicating an interaction between inherited variation and the growth changes due to epigenetic programming. Lower methylation at the IGF2 DMR in infants supports the central role of imprinted growth regulators in fetal size determination. Given that IGF2 promotes cell proliferation and nutrient signaling, altered methylation at this locus may reduce growth signaling capacity (107). Together, these findings support IGF2 methylation as a sensitive marker of fetal growth variation, but also underscore the complexity introduced by genetic background.

Similarly, a study of 100 placental biopsies, paired with maternal and cord-blood samples, demonstrated that methylation in IGF2 DMRs DMR0 and DMR2 in the placenta was positively correlated with newborn anthropometric measures, including birthweight, as well as maternal circulating IGF2 concentrations in late pregnancy (108). On the contrary, methylation in the H19 DMR correlated with IGF2 levels in cord blood, suggesting locus-specific regulatory mechanisms. The maternal genotype in the IGF2/H19 polymorphism rs2107425 is further associated with birth weight, independent of maternal diabetes, glucose tolerance or BMI. Together, epigenetic and genetic variation at the IGF2/H19 locus explained approximately 31% of the variance in newborn weight, highlighting their central role in fetal growth. These results suggest that epigenetic modulation of imprinted growth factors can influence birth outcomes and may contribute to the developmental origins of the risk of later obesity, even in the absence of overt maternal metabolic disorders.

As an imprinted gene, IGF2 is expressed from the paternal allele, while the maternal allele is silenced through methylation at key DMRs, such as IGF2 DMR0 and the H19 DMR (109, 110). Proper maintenance of imprinting has been shown to be essential for balanced fetal growth and postnatal metabolic health. Disruptions in this imprinting process, including hypomethylation or loss of imprinting (LOI), lead to increased IGF2 expression and have been linked to increased adiposity, insulin resistance, and higher obesity risk later in life. Prospective studies have shown that reduced methylation in IGF2 DMRs in the umbilical cord blood is associated with increased childhood adiposity (100).

Maternal hyperglycemia can directly reprogram the fetal hepatic epigenome by altering insulin-IGF signaling and DNA methyltransferase activity. It has been shown in the mouse models of GDM that offspring exposed to intrauterine hyperglycemia exhibit hypermethylation at the Igf2/H19 DMRs in the liver, accompanied by reduced hepatic Igf2 and H19 expression (153). Igf2 is a key fetal growth factor that promotes insulin sensitivity and metabolic homeostasis, while H19 participates in growth regulation and epigenetic control of the locus. Increased methylation at the imprint control region of these disrupts normal parent-of-origin expression patterns, leading to diminished Igf2 signaling. Mechanistically, maternal hyperglycemia activates hepatic forkhead box O1 (FoxO1), a transcription factor responsive to insulin signaling, which upregulates Dnmt3A expression (154). Elevated Dnmt3A enhances de novo DNA methylation at the Igf2/H19 locus, stabilizing epigenetic repression. Functionally, these offspring develop hepatic insulin resistance, increased gluconeogenic gene expression, and impaired glucose tolerance, demonstrating that maternal glycemia can induce persistent, locus-specific epigenetic modifications in metabolic tissue that contribute to later metabolic dysfunction (126). In pancreatic islets, intrauterine hyperglycemia causes abnormal Igf2/H19 DMR methylation linked to impaired islet structure/function and even transgenerational glucose intolerance.

In rat models of GDM, intrauterine exposure to maternal hyperglycemia induces persistent epigenetic alterations in pancreatic islets that impair β-cell function. Offspring of streptozotocin-induced diabetic dams exhibit hypomethylation of CpG sites within the promoters of Cdkn2a and cyclin-dependent kinase inhibitor 2B (Cdkn2b) in isolated pancreatic islets. This reduction in promoter methylation is accompanied by increased expression of the cyclin-dependent kinase inhibitors that function as the key regulators of cell-cycle arrest, resulting in β-cell proliferation, islet cell mass reduction, and promotion of premature cellular senescence (112). Functionally, these offspring display impaired glucose tolerance, reduced insulin secretion, and features of β-cell dysfunction, despite normal early postnatal growth. These findings suggest that maternal hyperglycemia can reprogram cell-cycle regulatory genes in the endocrine pancreas through locus-specific DNA methylation changes, limit β-cell regenerative capacity, and increase susceptibility to type 2 diabetes later in life (155).

## Factors affecting DNA methylation changes during childhood

### Nutrition and the offspring's BMI

Although the prenatal and early postnatal periods represent important windows of epigenetic plasticity, it is also true that diet continues to influence DNA methylation profiles well into adolescence and adulthood. For example, dietary glycemic load and overall nutritional quality in adolescence have been associated with altered methylation of metabolic genes, such as WD repeat domain 27 (WDR27) and colony-stimulating factor 3 (CSF3), with downstream implications for insulin sensitivity and fat distribution (113). These findings indicate that the methylome remains responsive to dietary exposures beyond early development and suggest that dietary patterns during periods of rapid growth and pubertal transition may meaningfully shape metabolic health trajectories.

Although most of the evidence on leptin epigenetics centers on intrauterine exposures, growing data indicate that environmental and social factors early in life continue to shape LEP methylation during infancy. In a cohort study of 17-month-old children, shorter duration of breastfeeding and lower maternal education were associated with higher methylation of LEP in whole blood, suggesting that caregiving practices and socioeconomic conditions exert a lasting epigenetic influence beyond birth. Sex and growth-related differences were also observed, in which boys demonstrated lower LEP methylation and higher birth weight compared to girls and higher birth weight, BMI, and serum leptin concentrations, each of which was associated with reduced methylation levels. These findings suggest that LEP methylation is dynamically responsive to both biological constitution and modifiable postnatal environments, with breastfeeding emerging as a potentially protective factor against adverse methylation profiles (114).

Work from Chinese pediatric cohorts furthered the relevance of HIF3A methylation beyond childhood. Obese children exhibited significantly higher methylation at 2 intronic CpG sites, one of which was correlated with elevated alanine aminotransferase (ALT), suggesting a possible link between HIF3A epigenetic status and susceptibility to nonalcoholic fatty liver disease (115). Similarly, POMC, a central gene that regulates appetite, exhibits hypermethylation of the intron 2 to exon 3 in obese children, which disrupts enhancer activity and reduces gene expression. This functional variant, likely mediated by nearby Alu elements, represents one of the first mechanistic examples of DNA methylation that directly contributes to pediatric obesity susceptibility (150).

Several large-scale EWAS have expanded beyond candidate loci, identifying novel methylation sites related to early adiposity and metabolic outcomes. For example, neonatal methylation in vault RNA 2-1 (VTRNA2-1, *nc886*) was strongly associated with BMI in later childhood, and methylation signatures near keratinocyte proline rich protein (KPRP), SCL9A10, myosin light chain kinase 2 (MYLK2), prolactin releasing hormone receptor (PRLHR), matrix metallopeptidase 25 (MMP25), and glutamine-fructose-6-phosphate transaminase 2 (GFPT2) were associated with adiposity throughout early and mid-childhood, although with inconsistent replication across phenotypes and time points (95, 117). A European EWAS in preschool-aged children further identified hundreds of CpGs related to BMI, fat mass, and fat-free mass, implicating genes such as sushi, nidogen, and EGF like domains 1 (SNED1), kelch like family member 6 (KLHL6), CASP8 and FADD like apoptosis regulator (CFLAR), PR/SET domain 14 (PRDM14), SOS Ras/Rac guanine nucleotide exchange factor 1 (SOS1), and ST6 beta-galactoside alpha-2,6-sialyltransferase 1 (ST6GAL1), many of which are involved in glucose and lipid metabolism (118). Collectively, these findings suggest that both gene-specific and genome-wide methylation signatures contribute to early-life programming of risk of obesity.

Epigenetic modulation at imprinted loci has also been implicated in sex-specific growth and the risk of obesity. Data from the Newborn Epigenetic Study showed that methylation in paternally expressed 10 (PEG10) was positively associated with infant weight in men. On the contrary, MEST methylation was correlated with lower weight in women, independent of small-for-gestational-age status (119). These associations are biologically relevant, as both PEG10 and MEST are paternally expressed imprinted genes that regulate placental development and adipogenesis, processes central to fetal growth and energy homeostasis (156, 157). These observations underscore the role of parent-of-origin-specific methylation changes in influencing growth trajectories in a sex-dependent manner. Importantly, longitudinal studies are beginning to disentangle cause from consequence. Some methylation signatures, such as Nuclear Respiratory Factor 1 (NRF1) in saliva, predict risk of obesity years before onset, independent of baseline BMI, supporting a role as early biomarkers (158). However, large population-based studies in adults show that most BMI-associated methylation changes are downstream consequences of adiposity rather than causal drivers (120-122). However, selected loci such as sterol regulatory element binding transcription factor 1 (SREBF1) and ANRIL retain functional evidence of causal involvement, highlighting that while obesity can shape the methylome, early-life methylation in specific regions may actively contribute to disease trajectories.

As the intrauterine environment has emerged as a key determinant playing a critical role in shaping the epigenome of the offspring and maternal metabolic health, evidence from studies of bariatric surgery provides some of the most compelling demonstrations that improving maternal metabolic status before pregnancy can confer lasting benefits on offspring through epigenetic pathways. In a sibling cohort that compared one child who was born before maternal bariatric surgery (BMS) with another who was born after surgery (AMS), researchers observed striking epigenetic differences between the siblings. Genome-wide methylation studies have identified 5698 differentially methylated genes with pathways related to glucose regulation, vascular function, and inflammation enriched (159). These methylation differences were correlated with gene expression differences as well as metabolic indicators, including fasting insulin and HOMA-IR, suggesting that improvements in maternal metabolic health translate into a more favorable metabolic profile in the AMS offspring. Complementary investigations focusing on inflammation-related pathways identified alterations in interleukin-8 (IL-8) signaling, both at the level of DNA methylation and in circulating inflammatory markers such as C-reactive protein (160). Together, these findings reinforce the idea that normalizing maternal weight prior to conception can induce broad and functionally relevant changes in the offspring methylome, with potential long-term benefits for cardiometabolic health. Studies on maternal obesity and gestational dietary interventions have also highlighted the importance of maternal nutrition as a modulator of offspring epigenetic patterns. In the ROLO trial, the adhesion to a low glycemic index diet during pregnancy produced significant differences in cord-blood methylation accounting for 11% of methylome variance at more than 770 000 CpG sites with enrichment in cardiac and immune-related pathways (161). However, replication in an independent cohort was not achieved, and follow-up at age 5 did not reveal persistent differences in methylation between intervention and control children, although postnatal weight trajectories were associated with variations in salivary methylation (162). These results suggest that while prenatal diet interventions may transiently shape the methylome of the offspring, sustained postnatal environmental influences likely play a dominant role in maintaining or erasing such effects.

Beyond glycemic control, maternal lifestyle interventions during pregnancy can induce measurable epigenetic alterations in offspring. In the Obese Pregnant Women (TOP) study, 208 women with obesity were randomized to receive a combined diet and physical activity intervention. Genome-wide methylation profiling in cord blood identified 379 differentially methylated CpGs, enriched in pathways related to fatty acid metabolism and development of adipose tissue (163). Mediation analysis has suggested that intervention-induced improvements in lean mass were partly explained by differential methylation at the CpG sites annotated to DISC1 scaffold protein (DISC1) and HECT and RLD domain containing E3 ubiquitin protein ligase 2 (HERC2) genes, indicating that epigenetic modulation of genes involved in intracellular scaffolding, cytoskeletal organization, and ubiquitin-mediated protein turnover may contribute to skeletal muscle remodeling and hypertrophic adaptation (123, 124). Furthermore, methylation at 22 CpG was associated with BMI z-scores in early childhood, supporting the hypothesis that maternal lifestyle modification during pregnancy can epigenetically prime healthier growth trajectories in offspring.

Together, these findings point to the fact that methylation in key metabolic regulators, including PPARγ, POMC, hepatocyte nuclear factor 4 alpha (HNF4A), HIF3A, and imprinted loci such as PEG10, MEST, IGF2, and H19, interconnects with broader genome-wide methylation signatures to shape early growth and adiposity. This layered regulatory architecture also draws special attention to the value of integrating candidate gene and epigenome-wide approaches to identify robust epigenetic predictors of childhood obesity. Importantly, locus-specific methylation changes which are observed in POMC, IGF2/H19, RXRa, PLAG1-like zinc finger 1 (ZAC1), and NRF1 genes point to the potentially informative early-life biomarkers; several of these changes are influenced by maternal diet and perinatal exposure. In contrast to this, genome-wide studies often reveal broader methylation changes that are more closely associated with adiposity itself. Integration of these perspectives needs longitudinal, functional, and mechanistic studies to distinguish the epigenetic drivers from downstream correlates and determine which marks have predictive or actionable value within the DOHaD framework.

Consolidative strategies combining EWAS with genetic tools, such as Mendelian randomization, along with functional testing using CRISPR-based epigenome editing platforms, have begun to identify methylation sites that directly regulate the expression of metabolic genes. An example is the enhancer methylation near the FTO locus, which alters chromatin looping and modulates iroquois homeobox 3 (IRX3) expression, a transcription factor that regulates adipocyte differentiation and the risk of type 2 diabetes (164). This is also an illustration of the transition from descriptive associations toward mechanistic understanding, highlighting pathways in which epigenetic modifications may ultimately be the focus of intervention.

### Maternal glycemia

Maternal glycemia and insulinemic index also exert quantifiable changes on the methylation patterns of the offspring. In a randomized trial involving 172 women who were either overweight or obese, maternal insulin sensitivity and glycemic indices during pregnancy were correlated with the differential methylation levels at specific CpG sites in cord blood and were mapped to genes involved in fetal growth and insulin regulation (165). Although the effect of lifestyle intervention was found to induce non-consistent changes in methylation, these observations reinforce the importance of maternal metabolic physiology as a driver of epigenetic variation. Because of the small effect size, although the associations which were CpG-specific were difficult to verify, this also underscores the need for large longitudinal cohorts to clarify causality. Maternal exercise in mouse models induces CpG-specific reductions in DNA methylation at metabolic regulators such as Ppargc1α in offspring skeletal muscle, accompanied by improved mitochondrial function and insulin sensitivity, illustrating how maternal lifestyle can drive targeted yet modest epigenetic variation in metabolic tissues (77, 125).

Evidence suggests that methylation differences associated with GDM can persist beyond birth. Salivary methylation profiling in infants born to GDM mothers identified 50 CpG sites that remained differentially methylated from 8 weeks to 1 year, with several correlating with early fetal growth and anthropometric traits (166). A parallel finding has been reported in rodent models of gestational diabetes showing persistent methylation changes beyond birth. In a streptozotocin-induced GDM mouse model, offspring exhibited alterations in DNA methylation at the Igf2/H19 imprinting control region in liver and pancreatic tissue that persisted into adulthood, accompanied by impaired glucose tolerance and insulin resistance (126). Such persistence highlights the potential of accessible tissues as early biomarkers of metabolic risk. More broadly, maternal obesity, GDM, and excessive weight gain have been associated with enduring methylation changes in metabolic regulatory genes, including LEP, POMC, and NR3C1, where methylation changes could be detectable into childhood and consistent with intergenerational transmission of metabolic risk through epigenetic mechanisms (167, 168).

### Early-life DNA methylation status of the offspring

Analysis of genome-wide methylation measures has shown gender-specific variations in obesity susceptibility. In a longitudinal study of 553 Colombian children, lower *LINE-1* methylation predicted increases in BMI for age, skinfold thickness, and waist circumference over 30 months, but only among boys (169).

Genome-wide and immune-focused studies suggested a broader mechanistic insight. One of the EWAS studies of adolescents in the Penn State Child Cohort identified the enrichment of differentially methylated CpGs within those genes, which are suggested to be obesity-related genes, including methylation changes in SIM BHLH transcription factor 1 (SIM1), a gene fundamental to the regulation of hypothalamic appetite, that is positively associated with BMI percentile (127). In rats, maternal high-fat diet exposure alters DNA methylation in the offspring hypothalamus, with differentially methylated CpGs identified in genes regulating appetite and energy balance, including melanocortin pathway regulators that function upstream or downstream of Sim1 (170). Complementary studies which have been carried out in adolescents with obesity have shown that altered methylation in immune regulatory loci, including hypermethylation in ubiquitin-associated and SH3 domain-containing A (UBASH3A) and hypomethylation in the tripartite motif containing 3 (TRIM3), implicates epigenetic disruption of immune pathways in the metabolic-immune interplay characteristic of adolescent obesity (128).

Longitudinal studies further highlight the stability of specific methylation signatures throughout the developmental stages. In the case of PPARG coactivator 1 Alpha (PPARGC1A), 7 promoter CpG sites remained unchanged over a decade of follow-up, with methylation at 4 sites being of predictive value of adiposity independently of age, sex, pubertal timing, and lifestyle factors (129). One of these sites directly influenced the binding of the PBX homeobox 1 (PBX1)/homeobox A9 (HOX9) complex, providing a reasonable functional mechanism linking early established DNA methylation to pro-adipogenic transcriptional regulation.

Methylation of the PPARγ promoter, a nuclear receptor encoding a master regulator of adipocyte differentiation and lipid metabolism, has been associated with lower gene expression and impaired adipocyte function (132, 171). In the CHAMACOS birth cohort, PPARγ methylation was temporally stable throughout childhood, with higher methylation at birth and at age 9 associated with lower birth weight and reduced BMI z-scores, respectively (172), suggesting that early methylation marks at this locus may have lasting effects on growth trajectories. In rats, maternal protein restriction induced hypermethylation of the Pparγ promoter in offspring adipose tissue. This alteration persisted postnatally and was associated with altered fat deposition later in their life (58). Similarly, in mouse models, early-life nutritional stress has been shown to modify Pparγ promoter methylation in adipose tissue, with corresponding changes in gene expression and adipocyte development (132).

The accumulation of evidence in experimental and human cohorts supports the contribution of early-life epigenetic regulation to later adiposity. Methylation at the ANRIL promoter, located at the CDKN2A locus, has been associated with adiposity at 6 years of age, with replication in newborns, adolescents, and adults in multiple tissues, including adipose tissue (102). In vivo expression data and in vitro promoter mutagenesis provide functional support that ANRIL methylation may modulate adipogenesis directly. Similar longitudinal studies have implicated methylation at the PPARγ coactivator-1α (PPARGC1A) promoter in shaping adiposity trajectories between the ages of 9 and 14 (129). Longitudinal profiling studies have also revealed stable methylation at 7 CpG sites between the ages of 5 and 14, with 4 loci independently predicting adiposity and one of these influencing the binding of the pro-adipogenic PBX1/HOX9 complex (129). These findings suggest that certain methylation marks, which are established early in development, remain stable and biologically meaningful, and are of predictive relevance for long-term metabolic outcomes.

Epigenetic changes are also seen in genes involved in central appetite regulation and hepatic metabolism. POMC, the archetypal polypeptide precursor of hormones and neuropeptides, which is crucial for appetite suppression, shows epigenetic dysregulation in both animal studies and in human cohort studies. In the Ewha Birth and Growth cohort, methylation of POMC exon 3 in children aged 7 to 9 years was positively associated with HDL cholesterol, suggesting links to favorable lipid regulation. Methylation at the HNF4A promoter, a transcription factor that regulates glucose and lipid homeostasis in the liver, was shown to be associated with total and HDL cholesterol levels independent of BMI, supporting BMI-independent epigenetic influence. On the contrary, methylation of the melanocortin 4 receptor (MC4R) did not show significant associations in this cohort, although its established role in monogenic obesity underscores its relevance for energy balance (131). Collectively, these findings indicate that DNA methylation in central and hepatic metabolic regulators can influence lipid profiles and metabolic health before the appearance of obvious adiposity, reinforcing their potential as early biomarkers.

Cord blood also provides a valuable window into early metabolic programming. Cord-blood leptin, a proxy for neonatal adiposity, correlates with methylation at 247 CpG sites at multiple loci, including DnaJ heat shock protein family (Hsp40) member A4 (DNAJA4), transferrin receptor 2 (TFR2), member of the SMAD family member 3 (SMAD3), PLAG1 zinc finger (PLAG1), fibroblast growth factor 1 (FGF1), and HNF4A, many of which regulate growth and energy metabolism (133). Moreover, methylation at MC4R and HNF4A in cord blood has been shown to predict metabolic outcomes in mid-childhood. Specifically, hypomethylation of MC4R is shown to be associated with elevated triglycerides at ages 7 to 8. As opposed to this, hypomethylation of the HNF4A promoter was shown to be inversely correlated with triglyceride levels (173). Consistent with this, rodent studies show that early-life nutritional disturbances alter DNA methylation at the Hnf4a locus, modifying its expression and further metabolic gene regulation involved in lipid regulation, supporting a mechanistic link between HNF4A methylation and triglyceride metabolism (174). Together, these findings highlight the potential value of locus-specific methylation signatures detectable at birth for forecasting cardiometabolic trajectories in later childhood. By linking molecular function with longitudinal prediction, these studies also strengthen the view that epigenetic variations may serve both as a mediator and a biomarker of obesity risk within the DOHaD concept.

The genes involved in postnatal growth dynamics provide additional evidence of epigenetic involvement. Rapid postnatal growth, a well-established risk factor for later obesity, was associated with altered expression and methylation of tumor-associated calcium signal transducer 2 (TACSTD2) in both preterm and term-born cohorts. While genetic variation at this locus influenced expression and methylation, it did not independently predict fat mass, suggesting that TACSTD2 methylation may act more as a biomarker than a causal mediator of adiposity (134).

Candidate gene studies provide some of the earliest evidence that DNA methylation plays a role in susceptibility to obesity. For example, POMC, a key regulator of appetite and energy balance, shows a hypermethylated variant at the intron 2 to exon 3 boundary in obese children, which reduces transcription by disrupting P300 enhancer binding (135). This locus-specific methylation appears to arise from Alu element–driven disruption of methylation boundaries, representing the first DNA methylation variant directly associated with the risk of pediatric obesity. Epigenetic modulation of appetite-regulating genes extends beyond POMC, as in African American children; altered methylation of brain-derived neurotrophic factor (BDNF) promoters was correlated with reduced satiety responsiveness in obese girls, highlighting gene–sex interactions in appetite control (175). Consistent with this, rodent models demonstrate that early-life nutritional excess induces hypermethylation of the Pomc promoter and altered Bdnf methylation in the hypothalamus, leading to persistent changes in appetite regulation and adiposity (116). Other candidate loci, including IGF2 and cytochrome P450 family 27 subfamily B member 1 (CYP27B1), also show altered methylation in obese children, with hypomethylation of IGF2 and CYP27B1 hypermethylation each correlating with BMI, suggesting distinct contributions from growth factor and vitamin D pathways to metabolic programming (136).

The imprinted IGF2/H19 axis has received particular attention as a mediator of early adiposity. Cord-blood hypermethylation at the H19 ICR has been associated with a higher weight-for-age at 1 year (176). On the contrary, longitudinal analyses in adolescents found that higher methylation at the same locus was positively associated with subcutaneous fat distribution, although not with visceral adiposity (177). These findings suggest that IGF2/H19 methylation may bias fat deposition patterns rather than general adiposity. Parallel studies have identified additional perinatal epigenetic predictors. Methylation in RXRα in umbilical cord tissue has been shown to robustly predict fat mass at 9 years of age in independent cohorts, with maternal carbohydrate intake in early pregnancy being inversely related to methylation (100). Similarly, ZAC1 DMR methylation in cord blood was correlated with fetal weight, birth weight, and 1-year growth and was influenced by maternal alcohol and vitamin B2 intake, underscoring the sensitivity of fetal epigenetic programming to maternal nutrition (137).

**Figure 4:**
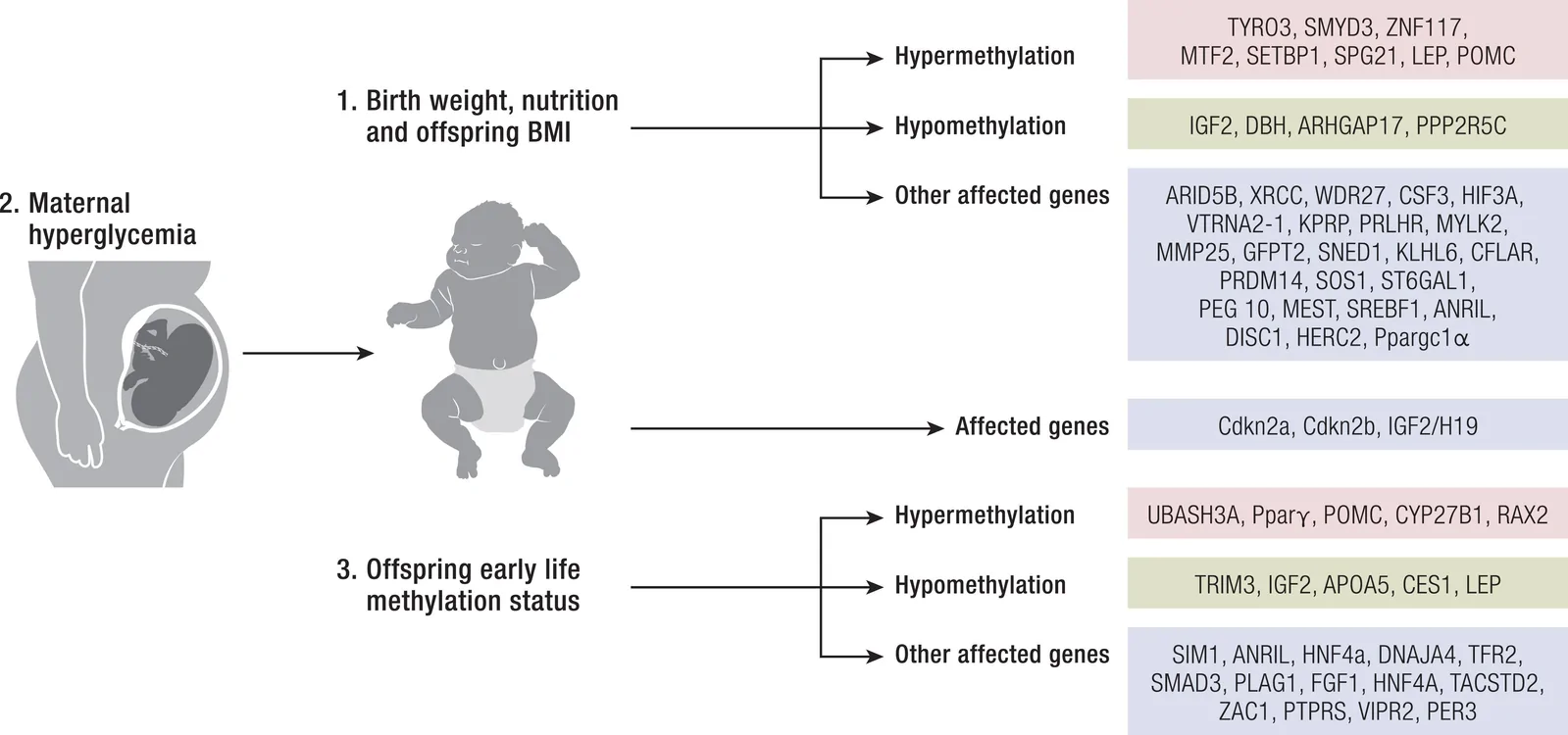
Postnatal factors shaping growth trajectories and associated DNA methylation patterns. Summary of key postnatal influences on DNA methylation during early childhood. Genes are grouped according to methylation status (hypermethylated, hypomethylated, or with uncertain/variable directionality), as indicated by the labels adjacent to each gene list.

Genome-wide methylation profiling in obese children has identified hundreds of differentially methylated CpG sites enriched in pathways related to immune function, lipoprotein metabolism, and circadian rhythm regulation (178-180). For example, methylation at HDAC4 and retina and anterior neural fold homeobox 2 (RAX2) was positively correlated with obesity, while apolipoprotein A5 (APOA5) and carboxylesterase 1 (CES1) were hypomethylated, reflecting altered lipid metabolism. Replicated findings also implicate FYN proto-oncogene, src family, tyrosine kinase (FYN), piwi-like RNA-mediated gene silencing 4 (PIWIL4), and tao kinase 3 (TAOK3), suggesting dysregulation of signaling and energy balance pathways (138). Large-scale EWAS have identified several hundred CpGs in children, with notable loci in type 1 protein tyrosine phosphatase receptor (PTPRS) and circadian genes (vasoactive intestinal peptide receptor 2 [VIPR2], period circadian regulator 3 [PER3] linking methylation changes with diet-driven energy regulation) (139, 140). The postnatal factors shaping growth trajectories and associated DNA methylation patterns are summarized in Fig. 4.

## Factors affecting DNA methylation changes during adolescence

### Nutrition and BMI

Adolescence represents a critical developmental window during which epigenetic modifications can consolidate early-life programming and interact with hormonal, metabolic, and environmental changes to influence the risk of obesity. Candidate gene studies have underscored the ongoing importance of leptin regulation. In a cohort of 431 adolescents, LEP promoter methylation, measured in saliva, was inversely associated with BMI z-score, waist circumference, and body fat percentage; however, this relationship was observed only in obese boys (141). Consistent with human adolescent findings, mouse models of diet-induced obesity demonstrate altered Lep promoter methylation in adipose tissue, correlated with leptin expression and adiposity, indicating that leptin regulation is epigenetically sensitive to metabolic state in vivo (181). These findings suggest that the epigenetic regulation of LEP contributes to the sex-specific trajectories of adiposity, further emphasizing saliva as a practical and noninvasive tissue for the discovery of adolescent epigenetic biomarkers.

Several candidate gene studies have shown that methylation of LEP and ADIPOQ, 2 key adipokines that regulate energy balance and insulin sensitivity, is sensitive to obesity and insulin resistance status in youth. In a cohort of 106 adolescents, obese individuals with insulin resistance exhibited significantly reduced methylation at specific CpG sites within both LEP and ADIPOQ promoters compared to lean peers and obese counterparts, but insulin-sensitive individuals (142). These changes were accompanied by elevated leptin and insulin levels and reduced adiponectin levels, highlighting the potential of LEP and ADIPOQ hypomethylation as early blood-based biomarkers of the risk of metabolic syndrome. Complementary findings showed that ADIPOQ promoter methylation in CpG-74 correlated inversely with BMI, triglycerides, glucose, and cholesterol, while CpG-283 hypomethylation was explicitly associated with insulin resistance, regardless of obesity status. Together, these results suggest that ADIPOQ methylation is epigenetically sensitive to systemic metabolic dysregulation and may provide an early indicator of the risk of cardiometabolic disease.

Epigenetic variation in imprinted genes also contributes to metabolic heterogeneity in obese adolescents. Studies of IGF2, a growth regulator sensitive to intrauterine conditions, have shown that the methylation status in obese youth stratifies individuals by lipid profile. Those with intermediate methylation exhibited significantly higher triglycerides and adverse triglycerides to HDL ratios compared to hypomethylated peers, suggesting that IGF2 methylation may serve as a marker of risk of lipid metabolism (143).

Beyond individual candidate genes, genome-wide studies highlight immune pathways and variability in methylation as hallmarks of adolescent obesity. Differential methylation in immune-related genes, including the ubiquitin-associated and SH3 domain-containing A (UBASH3A) (hypermethylated) and TRIM3 (hypomethylated), has been identified in both genome-wide screens and targeted follow-ups, suggesting that obesity-related immune dysregulation is at least partly epigenetically mediated (128, 144). Furthermore, analysis of differential variability CpGs (DVCs), which capture increased stochasticity in methylation patterns, revealed cancer-like epigenetic variability in obese adolescents, strengthening the notion that instability of the methylome may itself be a biomarker of disease susceptibility (144).

Meta-analytic approaches provide further evidence that obesity-related epigenetic patterns emerge during childhood and adolescence, and their magnitude increases with age. A large-scale meta-analysis of more than 4000 children identified three CpG sites (cg05937453, allograft inflammatory factor 1 gene; cg25212453, PR domain containing 16 gene; and cg10040131, neurexin 2 gene) that were significantly associated with BMI, with stronger effect sizes in older children, suggesting an age-dependent convergence toward adult-like methylation patterns (182). Similar results were reported in genome-wide studies of African American youth, where 76 CpG sites were robustly associated with obesity, independent of cell-type composition and metabolic confounders. Importantly, a subset of these CpGs was correlated with the expression of nearby genes, providing functional evidence that methylation changes in youth may contribute to altered transcriptional profiles relevant for adiposity and metabolic disease (183).

Epigenome-wide analyses in Chinese preschool and adolescent cohorts further reinforce the polygenic and systemic nature of obesity-related methylation. Hundreds of promoters were found to be differentially methylated in obese children, with enrichment in cancer-related and growth-regulatory pathways, including frizzled class receptor 7 (FZD7), prolactin-releasing hormone receptor (PRLHR), and eukaryotic translation initiation factor 6 (EIF6) (145). Consistent with these EWAS findings, mouse models of diet-induced obesity demonstrate widespread promoter methylation changes in the liver with enrichment in growth- and Wnt-related pathways, supporting the concept that obesity is associated with coordinated, polygenic epigenetic remodeling in metabolically active tissues (51). Candidate-driven analyses of those genes identified from obesity GWAS studies have shown consistent hypermethylation in lymphocyte antigen 86 (LY86), a gene implicated in inflammatory signaling, across multiple cohorts comprising more than 1000 adolescents. Methylation at the LY86 gene was correlated with adiposity, insulin resistance, and inflammatory status, linking genetic susceptibility, epigenetic modulation, and metabolic dysfunction (146).

**Figure 5:**
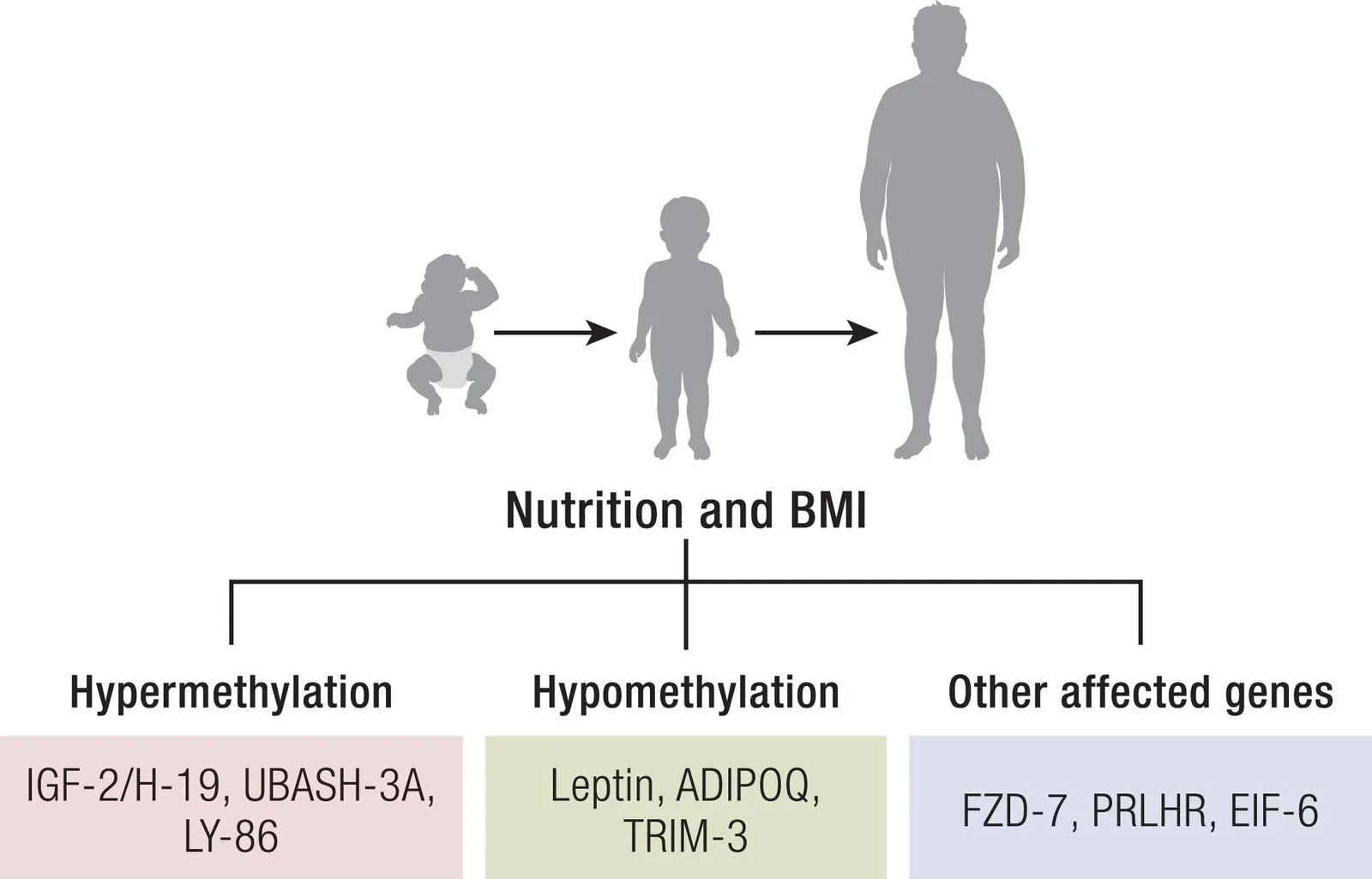
Factors influencing DNA methylation changes during adolescence. Figure summarizing the key DNA methylation alterations occurring during adolescence that modulate metabolic pathways and contribute to increased obesity risk during this developmental period. Genes are grouped according to methylation status (hypermethylated, hypomethylated, or with uncertain/variable directionality), as indicated by the labels adjacent to each gene group.

Together, these studies have established adolescence as an essential developmental window during which obesity-related methylation patterns not only consolidate themselves but also begin to exhibit measurable associations with systemic metabolic changes. Candidate gene analyses highlight the functional relevance of loci-specific methylation of LEP, ADIPOQ, and IGF2 genes in the shaping of adiposity, lipid metabolism, and insulin sensitivity. At the same time, genome-wide methylation analysis reveals a greater involvement in immune pathways, developmental signaling, and stochastic epigenetic variations. Notably, different loci identified also suggested functional correlations with gene expression and biochemical traits, suggesting that they may have potential value as biomarkers for early detection, monitoring and stratification of metabolic diseases, including obesity in the youth. Although this and candidate loci such as LEP, HIF3A, and POMC provide mechanistic information, global measures and EWAS findings emphasize the polygenic and system-level nature of adolescent epigenetic regulation. Furthermore, the presence of sex-specific and tissue-specific effects in multiple studies suggests that personalized epigenetic profiling may be necessary to refine risk prediction and guide targeted prevention strategies for adolescent obesity. The factors influencing DNA methylation changes during adolescence are shown in Fig. 5.

Similarly, longitudinal epigenome-wide analyses from birth to late adolescence have shown global DNA methylation patterns change markedly with increasing age, although some CpG sites exhibit stable changes which can be associated with early-life exposures (184). These studies suggest that a given epigenetic signature may reflect the biological susceptibility or altered physiological set points that interact with the cumulative postnatal exposures, such as nutrition, adiposity, stress, and other environmental factors, together influencing later disease risk (185, 186).

Hormonal and genetic changes occurring during puberty result in the activation of the hypothalamic-pituitary-gonadal axis. This is accompanied by physical growth and can reshape regulatory regions, which may reinforce, modify, or diminish earlier differences in DNA methylation (187, 188). Thus, human-specific developmental timing characterized by a prolonged prepubertal period exceeding a decade supports a model in which early epigenetic programming establishes an initial regulatory architecture that remains plastic and responsive to later environmental inputs through which their phenotypic consequences are shaped, amplified, or attenuated by subsequent exposures rather than being irreversibly fixed at birth.

The impact of postnatal DNA methylation changes on offspring adiposity is summarized in Table 1.

## Clinical implications and reversibility

### DNA methylation as a modifiable biomarker in childhood obesity

Epigenetic modifications, including DNA methylation marks associated with obesity, exhibit a degree of plasticity and can be modulated or partially reversed through targeted interventions such as diet modifications, physical activity, and metabolic surgery. These strategies, which produce significant clinical improvements in weight and metabolic health, have been shown to influence the epigenome, suggesting an avenue for long-term improvements in disease risk.

Weight loss achieved through caloric restriction or bariatric surgery can partially normalize methylation at genes related to inflammation and metabolism, PPARG coactivator 1 alpha (PGC1α), interleukin 6 (IL6), and tumor necrosis factor (189, 190).

Exercise induces favorable changes in methylation in skeletal muscle and liver, improving mitochondrial function and metabolic flexibility (191). Exercise interventions, alone or in combination with metabolic surgery, also cause epigenetic changes. Six months of aerobic and strength training in post-bariatric women has been shown to alter methylation at 722 CpG sites in whole blood, particularly genes related to Th17-cell differentiation and inflammation (192). Similarly, a 14-week combined exercise program in older obese women reshaped blood methylation in 1043 CpGs, impacting lipogenesis and oxidative phosphorylation pathways but not altering epigenetic age (193). Exercise has also been shown to remodel methylation in skeletal muscle genes, such as PGC-1α, with long-lasting histone acetylation reported in trained individuals, suggesting broad epigenetic reprogramming across tissues (194).

Beyond lifestyle and surgical interventions, pharmacological treatments have also been suggested to modulate obesity-associated epigenetic patterns. Growth hormone (GH) therapy, commonly used to promote catch-up growth in children born small for gestational age (SGA), may influence long-term metabolic trajectories not only through endocrine mechanisms but also through epigenetic remodeling. In a case–control study of low birth weight children, those receiving GH treatment suggested that lower BMI and reduced triglyceride concentrations compared to untreated peers. Genome-wide methylation profiling revealed distinct differential methylation patterns between treated and untreated groups, including hypermethylation of genes, such as Homeobox A5 (HOXA5), RUNX family transcription factor 1 (RUNX1), and N-Acylsphingosine Amidohydrolase 1 (ASAH1), and hypomethylation of loci including Complement C3 (C3), Tripartite motif containing 38 (TRIM38), and ZDHHC Palmitoyltransferase 13 (ZDHHC13). These findings suggest that GH therapy may induce systemic epigenetic shifts affecting pathways related to growth regulation, immune function, and lipid metabolism (195).

Dietary factors also shape the methylome. Adolescents with high glycemic diets exhibit altered methylation in colony-stimulating factor 3 (CSF3) and WD repeat domain 27 (WDR27) genes linked to inflammation and insulin sensitivity (196). Dietary interventions have been at the forefront of reversing adverse methylation patterns. Controlled overfeeding trials have demonstrated that a 7-week (+750 kcal/day) high-fat or high-polyunsaturated fats (high-PUFA) diet induced differential DNA methylation in human subcutaneous adipose tissue (SAT), affecting more than 1700 genes, including POMC, FTO, INSR, and IL-6. However, only a few of these caused changes in gene expression after saturated fat overfeeding (eg, acyl-CoA oxidase 1 [ACOX1], FAT atypical cadherin 1 [FAT1], and none for PUFA), suggesting a disconnect between methylation and transcription in short-term exposures (197). Similarly, a 5-day high-fat diet in low-birthweight individuals triggered 652 CpG methylation in SAT, impacting genes involved in insulin signaling and glucose transport; however, expression changes were limited (198). These findings highlight that even brief dietary insults can rapidly reshape the methylome; the functional impact may be delayed or context dependent.

On the contrary, caloric restriction studies provide evidence that weight loss itself can reshape methylation marks in key metabolic genes. An 8-week hypocaloric intervention in overweight individuals produced methylation changes in ATPase phospholipid transporting 10A (putative) (ATP10A), WT1 transcription factor (WT1), aquaporin 9 (AQP9), CD44 molecule (IN blood group) (CD44), and dual specificity phosphatase 22 (DUSP22), with methylation patterns that predict the responsiveness to weight loss programs (181, 199). Similar benefits have been reported following a 6-month energy-restricted diet, which induced hypermethylation of phospholipase C eta 2 (PLCH2) and PR/SET domain 8 (PRDM8) in the subcutaneous adipose tissue (SAT) (200). Large-scale clinical studies provide further support for the modulatory impact of dietary intervention on the epigenome. In the CALERIE trial, moderate calorie restriction (25%) sustained over 2 years slowed epigenetic aging as measured by the DunedinPACE biomarker, although traditional methylation clocks such as PhenoAge and GrimAge showed minimal change (201).

Finally, isolated studies of multimodal interventions show promise. For example, adherence to plant-rich diets and regular physical activity are consistently correlated with reduced biological age as measured by methylation clocks (202). A 6-month integrated lifestyle program in middle-aged males also produced concordant global methylation and gene expression changes, although the specific loci involved varied (203).

Pharmacological agents targeting DNMT and other epigenetic enzymes are being explored for the treatment of metabolic diseases, although their systemic effects have not yet been fully characterized (204). In summary, although overfeeding rapidly induces adverse methylation patterns, interventions such as dietary restriction, physical activity, and metabolic surgery can stabilize or reverse these changes. However, the effects vary depending on the gene, tissue, and duration. Clinical interventions often produce modest but clinically significant epigenetic benefits, particularly when sustained. However, the complexity and heterogeneity of tissue-specific methylation responses necessitate longitudinal, multi-tissue, and omics-driven studies to identify the most effective therapeutic windows and develop epigenetic biomarker-guided interventions.

### DNA methylation, childhood obesity, and personalized medicine

The connection of obesity, DNA methylation, and personalized medicine is increasingly being recognized as a crucial process to our understanding and better management of the metabolic diseases in the 21st century. It is being increasingly recognized that obesity, including childhood obesity, is not a simple outcome of caloric imbalance or sedentary behavior; instead, it is a reflection of a complex interplay of genetic susceptibility and environmental exposures, which are in part mediated through epigenetic mechanisms, including DNA methylation (205). It may be of relevance that many methylation alterations precede clinical symptoms of the disease, positioning them as possible biomarkers for early risk stratification and targeted intervention, although they need to be verified for factors such as temporal stability, incremental predictive value, or pre-analytical standardization (206, 207). Methylation profiles also result from encapsulation of the cumulative physiological impact of exposures, including high-fat diets and other high-calorie nutrient intake, early-life undernutrition, and psychosocial stress, which conventional genetic testing does not capture at the early stages (202, 208).

Recent advances in high-resolution methylome sequencing techniques, combined with the availability of machine learning algorithms, enable the integration of methylation signatures of individuals with the respective clinical, genetic, and lifestyle data, which together can guide personalized interventions (209). Such approaches have the potential to inform the dietary, behavioral, or pharmacological strategies that are tailored to the epigenetic architecture of an individual. By embedding DNA methylation analysis in precision medicine frameworks, clinicians have the opportunity to enhance predictive precision, refine the timing of preventive interventions, and ultimately move toward targeted epigenetic therapies capable of reshaping disease trajectories at the molecular level, although this is still in its early stages (210, 211). Such an integrative model will shift the field beyond the conventional risk scoring toward a genuine archetype of precision prevention and treatment in metabolic diseases, including that of childhood obesity.

### Future directions and challenges

Although epigenetics, including DNA methylation, offers considerable potential for precision medicine, translating these insights into clinical practice remains a challenge. One of the major limitations is establishing causality. Although earlier studies have reported the associations between DNA methylation, histone modifications, and metabolic outcomes, determining whether these changes precede disease or result from downstream physiological disturbances is often very difficult to establish. Experimental designs which are capable of differentiating the temporal sequence of events remain limited (212), and many findings remain correlational rather than real mechanistic insights.

Interindividual variability in epigenetic profiles adds another layer of complexity. Age, sex, ethnicity, environmental exposure, early developmental conditions, and lifestyle behaviors all influence epigenetic modification, contributing to the inconsistent verification of the results across studies and populations. Such heterogeneity further complicates the efforts to develop applicable biomarkers or therapeutic targets which can be applied universally (213). Technical limitations also contribute to variations seen, including batch effects across the different types of methylation platforms which are being used, variations in the various data processing pipelines, and the source of the samples being used which are predominantly the peripheral tissues such as blood, which may not accurately represent epigenetic states in disease-relevant organs such as liver, brain, or visceral fat which also continue to hinder the reproducibility and interpretability (214).

Furthermore, there are different bioinformatic processing pipelines being employed, and this lack of standardization in and variations in reference datasets limit comparability between studies. Standardized multi-omics frameworks that integrate genomic, transcriptomic, proteomic, and metabolomic datasets with epigenetic information will offer a more meaningful, comprehensive perspective on disease mechanisms, including that of childhood obesity (215). Longitudinal methylation tracking of different population cohorts will also be needed to define causal pathways, reveal epigenetic aging trajectories, and clarify the temporal dynamics of disease progression (213). It has also been proposed that adipose tissue retains an “epigenetic memory” of prior obesity, preserving transcriptional and chromatin features long after weight loss, suggestive of a process for why individuals often experience rapid weight gain and metabolic relapse (216). This, in turn, also highlights the need to characterize the temporal stability and reversibility of epigenetic marks.

Intervention studies, which are both lifestyle-based and pharmacological, will be needed to determine whether modifying epigenetic patterns can meaningfully alter clinical outcomes. Although it is of relevance to state that targeted manipulation of individual methylation marks remains technically challenging, there are rapid advances which are being made. CRISPR/dCas9-based epigenome editing systems, including platforms such as CRISPRoff, that allow precise and reversible modification of epigenetic states, offering new opportunities to test causal mechanisms and evaluate their therapeutic potential (217, 218). CRISPR-based epigenome editing in human iPSCs in conditions such as Prader–Willi syndrome demonstrates the feasibility of restoring gene function through targeted epigenome modulation, although still challenges exist at the level of the whole organism (219). However, they offer promising tools for experimentally testing causality and assessing therapeutic potential. Large-scale reference initiatives, such as the NIH Roadmap Epigenomics Project, have provided important foundational datasets, but advancing this information toward clinical translation will require studies that test the functional relevance, the reversibility, and integration of population variability of epigenetic modifications in diverse human cohorts (214). Ultimately, progress will depend on integrating mechanistic insight with longitudinal and interventional evidence, enabling epigenetic discoveries to inform precision prevention and personalized treatment strategies for metabolic diseases.

## Methods

We searched the PubMed database to find articles from 1975 to 2025 that were relevant to our research question using the keywords along with Boolean operators (Epigenetics, DNA methylation, Childhood obesity, DNMTs, Epigenome-wide association studies (EWAS), Differentially methylated regions (DMRs), Metabolic programming, Early-life nutrition, adiposity, Gene-environment interactions, Obese environment, Obesity-specific biomarkers, Maternal obesity, Epigenetic therapy, Personalised medicine). We screened the titles and the abstracts of the results to check for relevance and further grouped them based on the developmental stages and epigenetic factors. A review of the full texts followed this, based on how closely they related to the topic, their scientific quality, and how well they addressed the key points of our research question.

## Abbreviations

5caC: 5-carboxylcytosine
5fC: 5-formylcytosine
5hmC: 5-hydroxymethylcytosine
5mC: 5-methylcytosine
ALSPAC: Avon Longitudinal Study of Parents and Children
AMS: after maternal bariatric surgery
BER: base excision repair
BMI: body mass index
BMS: before maternal bariatric surgery
CRISPR: clustered regularly interspaced short palindromic repeats
DALYs: disability adjusted life years
DMR: differentially methylated region
DNMTs: DNA methyltransferases
DOHaD: developmental origins of health and disease
ELGAN: extremely low gestational age newborns
EWAS: epigenome-wide association studies
GDM: gestational diabetes mellitus
ICR: imprinting control region
NAFLD: nonalcoholic fatty liver disease
NEST: newborn epigenetics study
PACE: pregnancy and childhood epigenetics
SAT: subcutaneous adipose tissue
TDG: thymine DNA glycosylase
TET: ten-eleven translocation
